# Gestational Diabetes Mellitus Among Asians – A Systematic Review From a Population Health Perspective

**DOI:** 10.3389/fendo.2022.840331

**Published:** 2022-06-16

**Authors:** Ling-Jun Li, Lihua Huang, Deirdre K. Tobias, Cuilin Zhang

**Affiliations:** ^1^ Department of Obstetrics and Gynaecology, Yong Loo Lin School of Medicine, National University of Singapore, Singapore, Singapore; ^2^ Department of Medical Statistics and Epidemiology, Sun Yat-sen University, Guangzhou, China; ^3^ School of Public Health, Harvard University, Boston, MA, United States; ^4^ Epidemiology Branch, Division of Intramural Population Health Research, Eunice Kennedy Shriver National Institute of Child Health and Human Development (NIH), Bethesda, MD, United States

**Keywords:** gestational diabetes mellitus, Asians, prevalence, diagnostic criteria, diagnostic guidelines, maternal health outcomes, offspring health outcomes

## Abstract

**Objective:**

Since Asians are particularly vulnerable to the risk of gestational diabetes mellitus (GDM), the lifecourse health implications of which are far beyond pregnancy, we aimed to summarize the literature to understand the research gaps on current GDM research among Asians.

**Methods:**

We systematically searched the articles in PubMed, Web of Science, Embase, and Scopus by 30 June 2021 with keywords applied on three topics, namely “GDM prevalence in Asians”, “GDM and maternal health outcomes in Asians”, and “GDM and offspring health outcomes in Asians”.

**Results:**

We observed that Asian women (natives and immigrants) are at the highest risk of developing GDM and subsequent progression to type 2 diabetes among all populations. Children born to GDM-complicated pregnancies had a higher risk of macrosomia and congenital anomalies (i.e. heart, kidney and urinary tract) at birth and greater adiposity later in life.

**Conclusion:**

This review summarized various determinants underlying the conversion between GDM and long-term health outcomes in Asian women, and it might shed light on efforts to prevent GDM and improve the lifecourse health in Asians from a public health perspective.

**Systematic Review Registration:**

Prospero, CRD42021286075.

## Introduction

Diabetes is a significant cause of morbidity, mortality, and healthcare costs worldwide (1). The global age-adjusted comparative prevalence of diabetes among adults between 20-79 years of age was estimated at 8.3% (463 million) in 2019 (2), including 223 million women living with diabetes. And it is projected to reach 700 million people and 343 million women alone in 2045, respectively (2). Diabetes in pregnancy is similarly increasing in prevalence, with concerning consequences for both mother and offspring (3). Approximately 1 in 6 live births is affected by diabetes in pregnancy, 84% of which are diagnosed as gestational diabetes mellitus (GDM) (2, 4).

GDM is defined as glucose intolerance with the first onset or recognition during pregnancy (2, 4). Women with GDM have higher risks of cardiometabolic disorders during pregnancy and later in life (5). At the same time, offspring born to women with a history of GDM also encounter increased risks of developing obesity and other cardiometabolic disorders later in life (6, 7). The documented prevalence of GDM varies substantially worldwide, ranging from 1% to >30% (3), while compelling evidence has shown Asians share a high prevalence (i.e., Middle East: 8.8-20.0%; South-East Asia: 9.6-18.3%; Western Pacific: 4.5-20.3%) (3) regardless of the racial/ethnic differences in body mass index (BMI).

A meta-analysis found a more than sevenfold increased risk of T2DM in women with GDM after index pregnancy, compared with women with normoglycaemic pregnancies (8). Data on risk factors—particularly modifiable risk factors that may inform effective intervention strategies are relatively more collected in the Western population (e.g., North America, Europe, and Oceania) than the Asian population (3, 8–10). Research reporting a full spectrum of long-term health outcomes among both mothers and offspring following pregnancies complicated by GDM also mainly stemmed from the Western population (11). Furthermore, GDM studies have not been comprehensively reviewed on Asian immigrants exclusively, given that an increasing number of Asian migrants live in Western countries for a long-term residency (12). Due to the different environmental exposures such as socioeconomic transitions, lifestyle adaptations, cultural assimilation hardship, and health disparities^9,10^, there might be exceptionally high attributable risks on GDM development for Asian immigrants compared with Native Asians.

This review sought to summarize the literature to understand research gaps and develop future research directions on Asian women with GDM from a population health perspective. Thus, our review serves the objectives to 1) comprehensively examine the epidemiology of GDM, its risk factors, and health consequences; and 2) identify areas for future research for public health interventions to prevent GDM and its health consequences.

## Methods

### Search Strategy and Selection Criteria

We conducted the systematic review according to PRISMA for systematic review protocols. References for this review were identified through searches of Pubmed, Web of Science, Embase, and Scopus for articles published until 30 June 2021. We included three topics in our review, namely “Topic 1—GDM prevalence in Asians”, “Topic 2—GDM and maternal health outcomes in Asians”, and “Topic 3—GDM and offspring health outcomes in Asians”. Search terms included “prevalence”, “incidence”, “gestational diabetes mellitus”, “gestational diabetes” and “diabetes in pregnancy” in combination with the terms “Asia”, “Asians” and “Asian countries” in Topic 1. Search terms included “gestational diabetes mellitus”, “gestational diabetes” and “diabetes in pregnancy” in combination with the terms “Type 2 diabetes”, “prediabetes”, “glucose intolerance”, “abnormal glucose”, “hypertension”, “high blood pressure”, “cardiovascular disease”, “kidney disease”, “cancer”, “liver dysfunction”, “non-alcoholic fatty liver disease” and “health outcomes” and also in combination with the terms “After delivery” and “postpartum” in Topic 2. Search terms included “gestational diabetes mellitus”, “gestational diabetes”, “diabetes in pregnancy” and in combination with terms “cardio-metabolic outcome”, “cognitive outcome”, “congenital disease”, “adiposity”, “hypertension”, “health outcome”, “neuro-cognitive outcome”, “obesity”, “diabetes”, “cardiovascular disease”, “kidney disease” and “cancer” and also in combination with “child” and “offspring” in Topic 3. Articles resulting from these searches and relevant references cited in those articles were reviewed, among which reporting non-Asian human subjects or without full-text available were excluded. Flow charts for literature searching on each topic are shown in **Supplementary Figures 1**–**3**. The Prospero registration number for this systematic review is registered as CRD42021286075.

### Data Screening & Assessments

Double literature screening was conducted during the literature searching phase by two investigators (H L & L-J L). Furthermore, one investigator (A C) performed the quality assessments for all papers based on the Newcastle–Ottawa Scale Criteria (NOSC), and the other investigators (L-J L) verified the findingsindependently. The maximum score of 9 points in the Newcastle–Ottawa Scale is distributed in three aspects, namely selection of study groups (four points), comparability of groups (two points), and ascertainment of exposure and outcomes (three points) for case–control and cohort studies (13). We used the points to further categorize the publication quality with low risk of bias (between 7-9 points), high risk of bias (between 4-6 points), and very high risk of bias (between 0-3 points) (**Supplementary Tables 1**, **2** **)**.

## Results

### Prevalence of GDM by Geography

#### Overview

**Figure 1:**
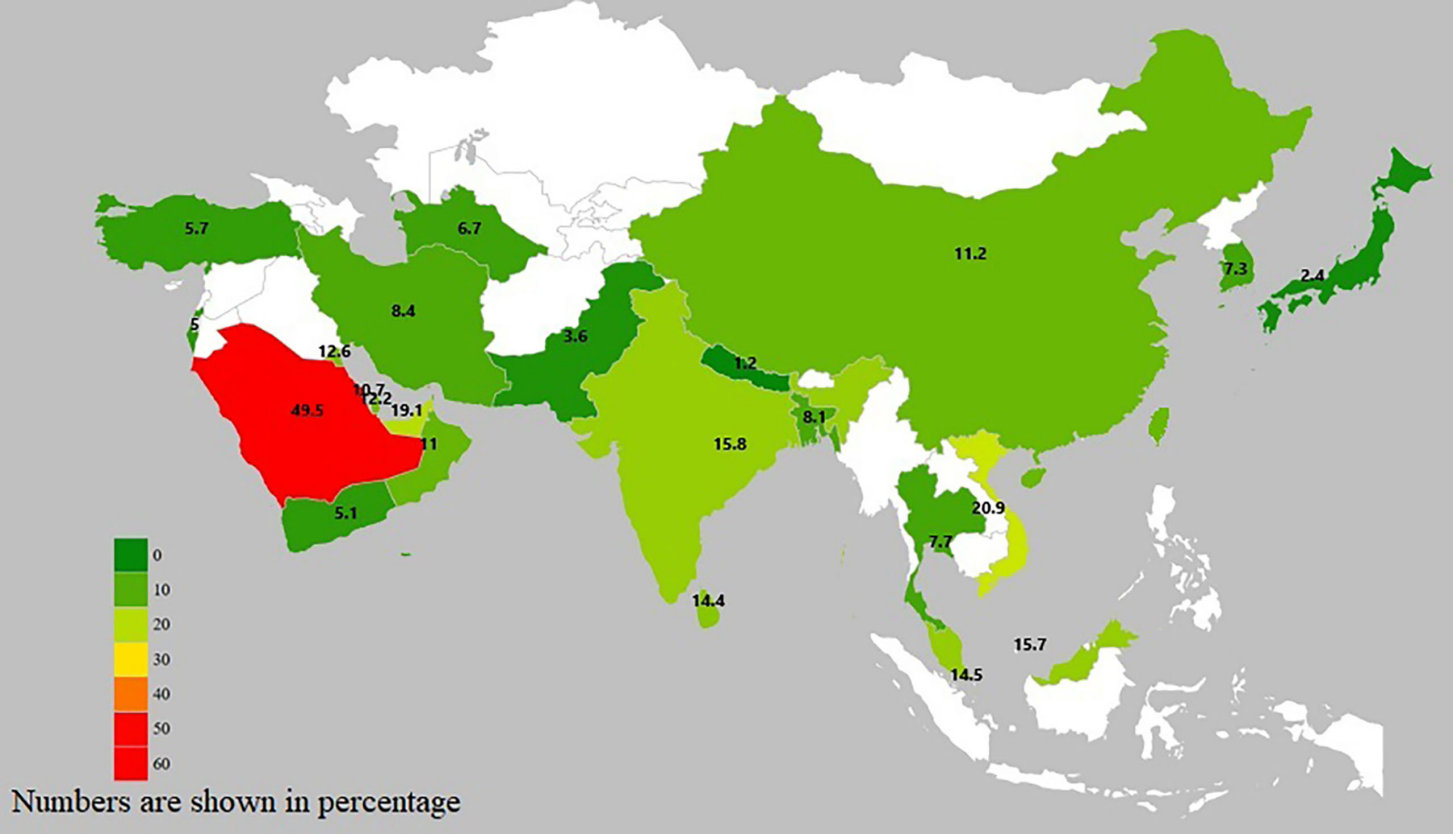
Asian geographic heat map on GDM prevalence.

GDM prevalence in Asian countries ranges widely from 1.2 to 49.5%, largely accounting for differences in diagnostic criteria, sample size and population source (e.g., hospital-based, community-based) (**Figure 1** and **Supplementary Table 3**).

#### Guideline-Specific Prevalence of GDM

**Figure 2:**
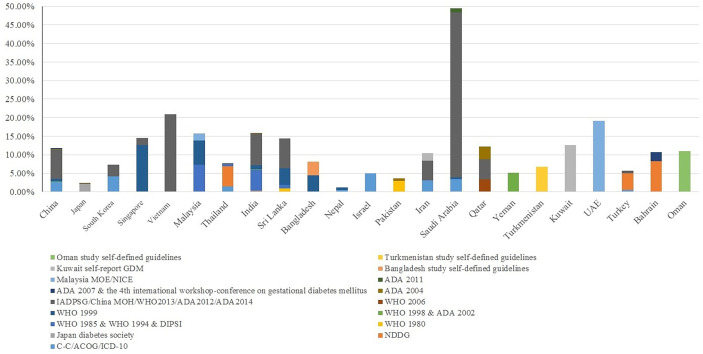
Country-specific prevalence of GDM in Asian studies. Due to the homogeneity of Chinese population residing in China, Taiwan and Hong Kong, we reported the country-specific prevalence of these three regions as a whole.

The prevalence of GDM varied substantially across Asian countries using different guidelines (**Figure 2**). We identified 29 GDM diagnostic criteria (**Supplementary Table 4**), among which the International Association of Diabetes and Pregnancy Study Groups (IADPSG) (14), World Health Organization (WHO) (15), Carpenter-Coustan (16), and American College of Obstetricians and Gynecologists (ACOG) (17) criteria were commonly used. Some countries adopted international guidelines as their national guidelines [e.g., China MOH guidelines (18), Malaysia MOH guidelines (19)], while some countries defined their own [e.g., Japan [Japan Diabetes Society] (20), India [Diabetes in Pregnancy Study group of India; DIPSI] (21), Turkmenistan (22), Oman (23)]. As the majority (123 out of 147) of included studies were published since 2010, we were not able to tease out whether the increment in GDM prevalence over the years in Asians is due to emerging evidence or new adoption of universal screening [i.e., IADPSG (14)].

**Figure 3:**
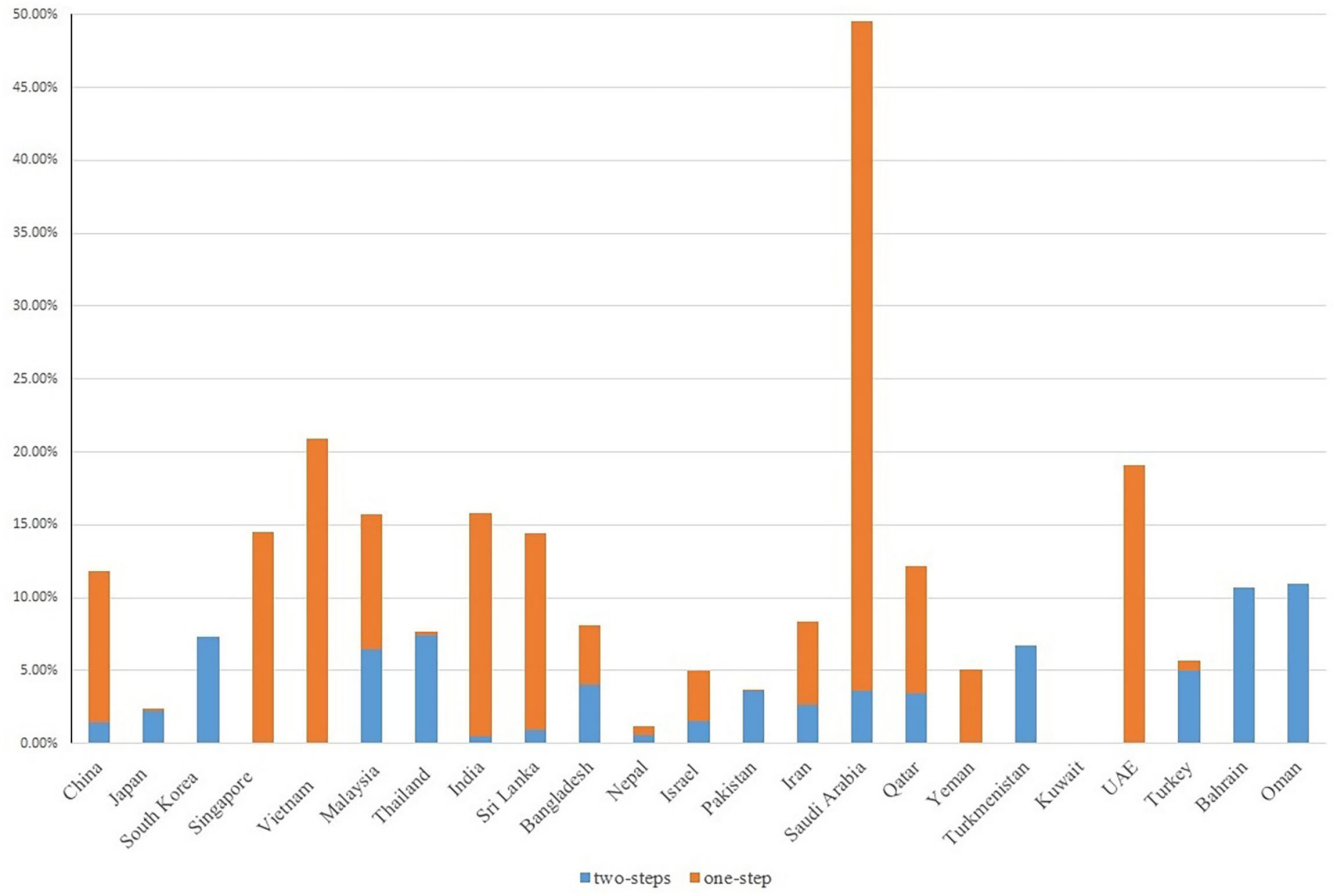
GDM screening steps with GDM prevalence in Asian studies. Due to the homogeneity of Chinese population residing in China, Taiwan and Hong Kong, we reported the country-specific prevalence of these three regions as a whole.

We included studies using either one-step or two-step diagnostic guidelines, the latter of which performed a 1-h 50-g glucose challenge test (GCT) glucose challenging test (GCT) additionally during 24-28 weeks of gestation, with a whole blood glucose threshold of 7.2 mmol/l (130 mg/dl). In general, we observed a link between adopting any one-step diagnostic guidelines (e.g., the IADPSG guidelines, the WHO 1999 guidelines) and higher GDM prevalence among Asian studies. For example, countries exclusively using (e.g., Singapore, UAE) or primarily using (e.g., China, Saudi Arabia, India) a one-step diagnostic approach reported an overall GDM prevalence above 10%. In contrast, countries exclusively using (e.g., Pakistan, South Korea) or primarily using (e.g., Thailand, Turkey, Japan) a two-step diagnostic approach reported an overall GDM prevalence below 10% (**Figure 3**).

#### Prevalence of GDM in Asian Migrants

Twenty-eight studies reported GDM prevalence among Asian migrants in Europe, Oceania, and North America, with sample sizes ranging from 1,491 to 10,823,924 participants. Overall GDM prevalence among Asian migrants is comparable to the Native Asian population. However, the prevalence of GDM was generally higher in Asian immigrants (0.18%-24.2%) than non-Hispanic White (NHW) (0.02%-7.0%) living in the same country, regardless of GDM diagnostic guidelines used (**Supplementary Table 5**). Among Asian immigrants in UK and Norway, South, East, and West Asian immigrants, as a whole, had doubled the odds for GDM than NHW (24, 25). Interestingly, length of immigration and birth countries seemed to relate to GDM prevalence. For instance, Danish-Chinese migrants with a longer stay (≥ 10 years) had a 62% higher odds of GDM onset than those with a shorter stay (≤ 5 years) (26). Also, foreign-born US-Indian migrants had a higher GDM prevalence than local-born US-Indian migrants (22.9% vs. 12.8%) (27).

### Adverse Health Outcomes and Attributable Risk Factors Following an Index GDM-Complicated pregnancy

#### Overview

**Figure 4:**
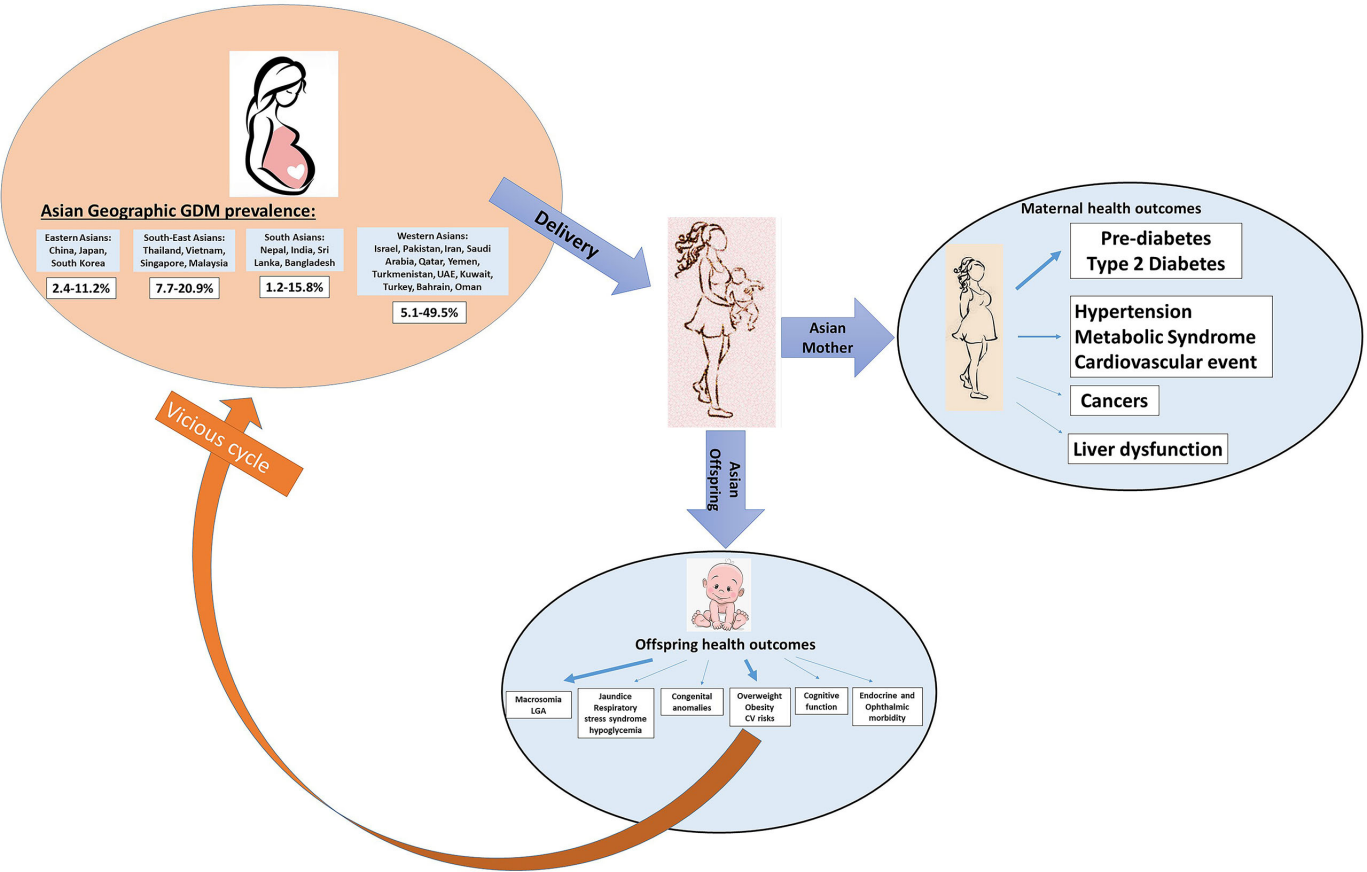
Schematic graphs of GDM leading transgenerational health outcomes in Asian studies. The arrow represents the found associations between GDM and different transgenerational outcomes. A thicker arrow indicates a higher number of studies reported on this topic.

Overall, seventy-two studies, predominantly longitudinal cohorts on GDM and maternal postpartum health outcomes, were identified in Asian countries (**Table 1** and **Figure 4** **)**. Among them, prediabetes and T2D, cardiovascular disorders, cancer, and non-alcoholic fatty liver disease (NAFLD) were reported following index pregnancy complicated by GDM, with a mean or median follow-up from 4 weeks to 38 years after delivery. The majority of studies were reported from East Asia (42/72 studies, 58.3%), especially in the Chinese population. Two studies that reported postpartum T2D development in Asian immigrants were identified (**Supplementary Table 6**). Thirteen out of 74 included studies (18%) were assessed low in risk of bias, while the rest majority (80%) were either high or very high risk of bias (**Supplementary Table 1**).

**Table 1:** Summary of studies addressing GDM-related maternal health outcomes in Native Asians.

Maternal Health Outcome	Country	No	PMID	Author	Year	Study design	Mean or range of follow-up	No of GDM	No of outcome cases	Cumulative incidence rate; Incidence rate (per 1000 person-years) if applicable*	Baseline age, years	Baseline BMI, kg/m^2^	GDM diagnosis guidelines	Outcome diagnostic guidelines
Pre-diabetes and T2D	China	1	33036614	Pei et al.,	2021	Retrospective cohort study	6-12 weeks	589	Pre-diabetes: 191 T2D: 18	Pre-diabetes: 32.4% T2D: 3.1%	33-34 (follow-up)	21.49-21.99	IADPSG	WHO 1999
		2	32515856	Mao et al.,	2020	Cross-sectional	1.5 year	425	Pre-diabetes: 62 T2D: 27	Pre-diabetes: 14.6%; 97 T2D: 6.3%; 42	32.3	>24: 69.2% 24-27.9: 24.7% ≥28: 6.1%	Did not define	WHO 1999
		3	32080127	Miao et al.,	2020	Prospective cohort	5.5 years	55	Pre-diabetes: 19 T2D: 9	Pre-diabetes: 34.6%; 63 T2D: 16.4%; 30	31	22.5	NDDG & IADPSG	WHO 1999
		4	31179619	Wang et al.	2019	Prospective cohort	6-12 weeks	583	Pre-diabetes: 157 T2D: 17	Pre-diabetes: 26.9%; N.A. T2D: 2.9%; N.A.	32.5	<25: 78.0% ≥25: 22.0%	Chinese MOH	WHO 1998
		5	30999888	Liu et al.,	2019	Prospective cohort	6 months	91	Pre-diabetes: 27 T2D:	Pre-diabetes: 29.7%; N.A. T2D: 1.1%; N.A.	32.7	<18.5: 16.0% <18.5-24.9: 69.6% ≥25.0: 14.3%	IADPSG	WHO 1999
		6	31472162	Fan et al.	2019	Prospective cohort	4.22 years	1263	Pre-diabetes: 457 T2D: 114	Pre-diabetes: 36.2%; 86 T2D: 9.0%; 21	32.4	23.1	WHO 1999	WHO 1999
		7	30182781	Ma et al.,	2018	Prospective cohort	6-8 weeks	472	Pre-diabetes: 121 T2D: 57	Pre-diabetes: 25.6%; N.A. T2D: 12.1%; N.A.	31.3	23.1	IADPSG	WHO 1999
		8	24397392	Mai et al.,	2014	Case-control	2.5 years	190	T2D: 19	T2D: 10%; 40	33.1	22.7	ADA 2004	ADA 2010
		9	25271112	Chang et al.,	2014	Prospective cohort	6 weeks ~ ≥ 1 year	282	T2D: 8	T2D: 2.8%; N.A.	29.6	26.2	ADA 2007	did not define
		10	18701021	Cao et al.,	2008	Prospective cohort	6-8 weeks	186	Pre-diabetes & T2D: 52	Pre-diabetes & T2D: 28.0%; N.A.	32.1	21.9	WHO 1999	WHO 1999
	Taiwan	11	25865283	Lin et al.,	2016	Retrospective cohort study	6 months - 9 years	71	T2D: 29	T2D: 40.8%; N.A.	31.7	24.9	NDGG	ICD
	Hong Kong	12	23897066	Shek et al.,	2014	RCT	36 months	170	T2D: 9	T2D: 5.3%; 18	39	24.4	WHO 1999	WHO 1999
		13	22179684	Tam et al.,	2012	Prospective cohort	15 years	45	Pre-diabetes: 12 T2D: 11	Pre-diabetes: 26.7%; 18 T2D: 24.4%; 16	43.8 (follow-up)	24.7 (follow-up)	WHO 1999	WHO 1999
		14	21636867	Lee et al.,	2011	Prospective cohort	52 months (4.3 years)	238	T2D: 47	T2D: 19.7%; 46	33.9	24.9 (follow-up)	WHO 1998	WHO 1998
		15	10687769	Ko et al.,	1999	Prospective study	6 weeks	801	Pre-diabetes: 182 T2D: 105	Pre-diabetes: 22.7%; N.A. T2D: 13.1%; N.A.	34	24.8	Abell and Beischer criteria *	WHO 1985
	Japan	16	31969529	Kawasaki et al.	2020	Retrospective cohort study	1 year	399	T2D: 43	T2D: 10.8%; N.A.	34.1	23.4	JSOG/IADPSG	ADA 2019
		17	30239167	Kasuga et al.,	2019	Prospective cohort	24.9 weeks	213	Pre-diabetes: 51 T2D: 8	Pre-diabetes: 23.9%; N.A. T2D: 3.8%; N.A.	37	21.6	IADPSG	JSOG
		18	29596944	Inoue et al.	2018	Retrospective cohort study	2 years	77	Pre-diabetes: 17 T2D: 17	Pre-diabetes: 22.1%; 110 T2D: 22.1%; 110	34.3	23.9	IADPSG	WHO 1998
		19	29706019	Kondo et al.,	2018	Retrospective cohort study	8-12 weeks	123	Pre-diabetes: 41 T2D: 4	Pre-diabetes: 33.3%; N.A. T2D: 3.3%; N.A.	34	21.4	IADPSG	WHO1999
		20	29310607	Kugishima et al.,	2018	Retrospective cohort study	1.09 years	306	T2D: 32	T2D: 10.5%; 96	33	23.5	JSOG/IADPSG	WHO 1999
		21	29624902	Nishikawa et al.,	2018	Prospective cohort	6-12 weeks	185	Pre-diabetes: 22 T2D: 3	Pre-diabetes: 11.9%; N.A. T2D: 1.6%; N.A.	33.05	23.15	IADPSG	ADA 2017
		22	28725256	Yasuhi et al.,	2017	Retrospective cohort study	1 year	88	Pre-diabetes: 29 T2D: 13	Pre-diabetes: 33.0%; N.A. T2D: 14.8%; N.A.	33.3	23.9	JSOG/IADPSG	WHO 2006
		23	25497883	Kugishima et al.,	2015	Retrospective cohort study	6-8 weeks	169	Pre-diabetes: 52 T2D: 6	Pre-diabetes: 30.8% T2D: 3.6%	32.6	23.5	JSOG/IADPSG	WHO 1999
	South Korea	24	30486265	Han et al.,	2018	Retrospective cohort study	10 years	4970	T2D: 470	T2D: 9.5%; 9	28.3	21	ICD-10	ICD-10
		25	27583868	Cho et al.,	2016	Prospective cohort	3.98 years	412	T2D: 51	T2D: 12.4%; 31	30.6	23.5	NDGG	ADA 2010
		26	27159192	Cho et al.,	2016	Prospective cohort	8 years	2962	T2D: 249	T2D; 8.4%; 11	29.9	21.7	ICD-10	ICD-10
		27	26996814	Kim et al.,	2016	Prospective cohort	6-12 weeks	699	Pre-diabetes: 343 T2D: 36	Pre-diabetes: 49.1%; N.A. T2D: 5.2%; N.A.	33	22.6	CC	ADA 2014
		28	26674320	Shin et al.,	2016	Prospective cohort	6-12 weeks	498	Pre-diabetes: 157 T2D: 40	Pre-diabetes: 31.5%; N.A. TD: 8.0%; N.A.	33.3	23.7	CC	ADA 2004
		29	26713061	Cho et al.,	2015	Retrospective cohort study	6-12 weeks	757	Pre-diabetes: 334 T2D: 139	Pre-diabetes: 44.1%; N.A. T2D: 18.4%; N.A.	33.7	23.7	CC	ADA 2011
		30	26171796	Moon et al.,	2015	Prospective cohort	4 years	283	T2D: 48	T2D: 17.0%; 42	32	23.3	NDGG	ADA 2010
		31	24431910	Yang et al.,	2014	Prospective cohort	15.6 months (1.3 years)	116	Pre-diabetes: 59 T2D: 8	Pre-diabetes: 50.9%; 39 T2D; 6.9%; 53	33.8	23.7 (follow-up)	NDGG	ADA 2011
		32	23471980	Kwak et al.,	2013	Prospective cohort	1 year	370	T2D: 88	T2D: 23.8%; N.A.	32	23	NDGG	ADA 2014
		33	24057154	Kwak et al.,	2013	Prospective cohort	3.75 years	395	T2D: 116	T2D: 29.4%; 78	31.4	23.2	NDGG	ADA 2013
		34	21106349	Kim et al.,	2011	Prospective	6-12 weeks	381	Pre-diabetes: 161 T2D: 27	Pre-diabetes: 42.3%; N.A. T2D: 7.1%; N.A.	34.2	23.6	CC	ADA 2004
		35	18456364	Lee et al.,	2008	Prospective cohort	2.1 years	620	T2D: 71	T2D: 11.5%; 55	33.6	23.5	NDGG	ICD
		36	17259506	Lim et al.,	2007	Prospective cohort	1 year	81	Pre-diabetes: 21	Pre-diabetes: 25.9%; N.A.	34 (follow-up)	22.9 (follow-up)	NDGG	Did not define
		37	16054264	Cho et al.,	2006	Prospective cohort	6 years	909	Pre-diabetes: 120 T2D: 116	Pre-diabetes: 13.2%; 22 T2D: 12.8%; 21	33.5 (follow-up)	24 (follow-up)	NDGG	NDGG
		38	12951280	Jang et al.,	2003	Prospective cohort	6-8 weeks	311	Pre-diabetes: 72 T2D: 47	Pre-diabetes: 23.2%; N.A. T2D: 15.1%; N.A.	30.9	22.7	Korean guidelines	WHO 1985
Pre-diabetes and T2D	Thailand	39	29926712	Ruksasakul et al.	2016	Case-control	2.97 years	56	Pre-diabetes: 29	Pre-diabetes: 51.8%; 174	38.6 (follow-up)	24.6	CC	ADA 2013
		40	23692133	Youngwanichsetha et al.,	2013	Cross-sectional	6 weeks	210	Pre-diabetes: 56	Pre-diabetes: 26.7%; 267	34.5	18.5-24.9: 23.8% 25-29.9:58.6% 30-39.9:17.6% (follow-up)	ADA 2010	ADA 2011
	Malaysia	41	23268155	Chew et al.,	2012	Cross-sectional study	84 months (7 years)	342	T2D: 53	T2D: 15.5%; 22	34.7	27.5 (follow-up)	WHO 1985	WHO 2002
	Singapore	42	33525398	Hewage et al.,	2021	Prospective cohort	1 year	116	Pre-diabetes: 38 T2D: 13	Pre-diabetes: 32.8%; 38 T2D: 11.2%; 11	33.3	23.7	WHO 1999	WHO 1999
	Philippines	43	N/A	Malong et al.,	2013	Prospective cohort	3 years	124	Pre-diabetes: 43 T2D: 9	Pre-diabetes: 34.7%; 116 T2D: 7.3%; 24	32.1	23.8	IADPSG/CC/WHO	ADA 2004
	India	44	29802954	Goyal et al.,	2018	Prospective cohort	20 months (1.7 years)	267	Pre-diabetes: 126 T2D: 28	Pre-diabetes: 47.2%; 278 T2D: 10.5%; 62	32.5	27.3	IADPSG	ADA 2014, WHO 2006
		45	27329018	Bhavadharini et al.,	2016	Prospective cohort	6 weeks -1 year	203	Pre-diabetes: 34 T2D: 7	Pre-diabetes: 16.7%; N.A. T2D: 3.4%; N.A.	29.1	26.9	IADPSG	ADA 2005
		46	26926329	Gupta et al.,	2017	Prospective cohort	14 months (1.2 years)	366	Pre-diabetes: 144 T2D: 119	Pre-diabetes: 39.3%; 328 T2D: 32.5%; 271	30.2	<25.0: 67.9% 25.0-29.9: 25.8% ≥ 30.0: 6.3%	IADPSG	ADA 2014
		47	25952037	Jindal et al.,	2015	Prospective cohort	6 weeks	62	Pre-diabetes: 17 T2D: 4	Pre-diabetes: 27.4%; N.A. T2D: 6.5%; N.A.	31.5	not specified	ADA 2011	ADA 2011
		48	24944938	Mahalakshmi et al.,	2014	Retrospective cohort study	4.5 years	174	T2D: 101	T2D: 58.0%; 129	29	28.6	CC	WHO 2006
		49	17640759	Krishnaveni et al.,	2007	Retrospective cohort study	5 years	35	Pre-diabetes: 11 T2D: 13	Pre-diabetes: 31.4%; 63 T2D: 37.1%; 74	28.2	25.5 (follow-up)	WHO 1999	WHO 2006
	Sri Lanka	50	29679628	Sudasinghe et al.,	2018	Prospective cohort	1 year	59	Pre-diabetes: 17 T2D: 11	Pre-diabetes: 28.8%; N.A. T2D: 18.6%; N.A.	<25: 8.9% 25-34: 58.0% 35-49: 33.1%	<18.5: 12.4% <18.5-24.9: 45.6% 25.0-29.0: 36.1% ≥30: 5.9%	WHO 1999	WHO 2006
		51	28644881	Herath et al.,	2017	Prospective cohort	10.9 years	119	T2D: 73	T2D: 61.3%; 56	31.7	<18.5: 1.5% 18.5-24.9: 57.4% ≥25.0: 41.1%	WHO 1999	WHO 1999
		52	16972862	Wijeyaratne et al.,	2006	Prospective cohort study	34.6 months (2.9 years)	147	Pre-diabetes: 56 T2D: 20	Pre-diabetes: 38.1%; 131 T2D: 13.6%; 47	33.4	26.3	WHO 1999	IDF
	Pakistan	53	28423981	Aziz et al	2018	Prospective cohort	2 years	78	Pre-diabetes: 3 T2D: 11	Pre-diabetes: 3.8%; 19 T2D: 14.1%; 71	28.9	not specified	IADPSG	Did not define
	Israel	54	31167664	Yefet et al	2019	Retrospective cohort study	15.8±5.1 years	446	T2D: 207	T2D: 46.4%; 31	30.1	27.0	CC and NDDG	ICD9
		55	20636958	Chodick et al.,	2010	Retrospective cohort study	5.7 years	11270	T2D: 1125	T2D: 10.0%; 18	32.7	<25: 14.6% 25-30: 16.7% >30: 20.0% unknown 48.6%	NDGG	MHS guidelines
	Turkey	56	24591906	Kerimoğlu et al.	2010	Prospective cohort	6-12 weeks	78	Pre-diabetes: 28 T2D: 27	Pre-diabetes: 35.9%; N.A. T2D: 34.6%; N.A.	31.3	27.7	CC	WHO 2006
	Iran	57	28432896	Minooee et al.	2017	Prospective cohort	12.1 years	476	Pre-diabetes: 279 T2D: 49	Pre-diabetes: 58.6%; 48 T2D: 10.3%; 9	36.5	28.4	WHO 1999	ADA 1997
		58	28491872	Nouhjah et al.,	2017	Prospective cohort	6-12 weeks	176	Pre-diabetes: 31 T2D: 8	Pre-diabetes: 17.6%; N.A. T2D: 4.5%; N.A.	29.7	27.8	IADPSG	ADA 2003
		59	25892996	Valizadeh et al.,	2015	Prospective cohort study	22.8 months (1.9 years)	110	Pre-diabetes: 11 T2D: 36	Pre-diabetes: 10%; 53 T2D: 32.7%; 172	>34:64.5% ≤34:35.5%	28.5	CC	Did not define
		60	17962102	Hossein-Nezhad et al.,	2009	Retrospective cohort study	6-12 weeks	114	Pre-diabetes: 24 T2D: 9	Pre-diabetes: 21.4%; N.A. T2D: 8.1%; N.A.	29	27.4	CC	ADA/WHO 1985
	UAE	61	15063951	Agarwal et al.	2004	Retrospective cohort study	4-8 weeks	549	Pre-diabetes: 114 T2D: 50	Pre-diabetes: 20.8%; N.A. T2D: 9.1%; N.A.	32	not specified	ADA 1997	WHO 1999
	Saudi Arabia	62	30186874	Wahabi et al.,	2018	Prospective cohort	1 year	133	Pre-diabetes: 60 T2D: 15	Pre-diabetes: 45.1%; N.A. T2D: 11.3%; N.A.	30.4	27.6	WHO 2013	ADA 2018
		63	31435382	Mahzari et al.,	2018	Retrospective cohort study	6 weeks	123	T2D: 82	T2D: 66.7%; N.A.	34	35.6	Did not define	Did not define
Cancer	South Korea	24	30486265	Han et al.,	2018	Retrospective cohort study	10 years	4970	Total cancer: 437 Thyroid Cancer: 131	Total cancer: 8.8%; 9 Thyroid Cancer: 2.6%; 3	28.3	21	ICD-10	ICD-10
	Taiwan	64	30796123	Peng et al.,	2019	Retrospective cohort	6.84 years	47373	Total cancer: 1063 Breast cancer: 284 Thyroid cancer: 91 Nasopharynx: 90 Lung and bronchus: 56 Kidney cancer: 25	Total cancer: 2.24%; 3 Breast cancer: 0.6%; 1 Thyroid cancer: 0.2%; 0.3 Nasopharynx: 0.2%; 0.3 Lung and bronchus: 0.1%; 0.2 Kidney cancer: 0.05%; 0.1	29.0	not specified	ICD-10	ICD-10
	Israel	65	28035489	Fuchs et al.	2017	Retrospective cohort	12 years	9893	Ovary cancer: 9 Uterine cancer: 11 Breast cancer: 91	Ovary cancer: 0.1%; 0.1 Uterine cancer: 0.11%; 0.1 Breast cancer: 0.919%; 1	31.8	1.1% with maternal obesity	Medical records	Medical records
		66	21847538	Sella et al.	2011	Retrospective cohort	5.19 years	11264	Digestive organ cancer: 13	Digestive organ cancer: 0.11%; 0.2	30.72	20.1% with maternal obesity	CC	Israel national cancer registry through linkage data
		67	17476589	Perrin et al.	2008	Retrospective cohort	34 years	410	Breast cancer: 29	Breast cancer: 7.1%; 2	<25-35+	Not specified	Medical records	Israel national cancer registry ICD-10
		68	17705823	Perrin et al.	2007	Retrospective cohort	38 years	410	Pancreatic cancer: 5	Pancreatic cancer: 1.2%; 0.3	<25-35+	Not specified	Medical records	Israel national cancer registry ICD-10
Hyperten-sion	Hong Kong	13	22179684	Tam et al.,	2012	Prospective cohort	15 years	45	Hypertension: 16	Hypertension: 35.6%; 24	43.8 (follow-up)	24.7 (follow-up)	WHO 1999	WHO 1999
	China	69	28660887	Wang et al.,	2017	Prospective cohort	2.29 years	1261	Hypertension: 94	Hypertension: 7.45%; 33	32,8	24.3	WHO 1999	2007 ESH, ESCG
		8	24397392	Mai et al.,	2014	Case-control	2.5 years	190	Hypertension: 10	Hypertension: 5.3%; 21	33.1	22.7	ADA 2004	ADA 2010
Dyslipidemia	China	1	33036614	Pei et al.,	2021	Retrospective cohort study	6-12 weeks	589	Dyslipidaemia: 227	Dyslipidaemia: 38.5%	33-34 (follow-up)	21.49-21.99	IADPSG	NCEP ATPIII criteria
Metabolic Syndrome (MetS)	China	70	30905596	Shen et al.,	2019	Prospective cohort	3.53 years	1263	Mets NCEP ATPIII criteria: 246 MetS by IDF criteria: 244	Mets by NCEP ATPIII criteria; 19.5%; 55 MetS by IDF criteria: 19.3%; 5473	30.1	24.2	WHO 1999	IDF, NCEP ATPIII criteria
		8	24397392	Mai et al.,	2014	Case-control	2.5 years	190	Mets: 38	MetS: 20%; 80	33.1	22.7	ADA 2004	ADA 2010
	South Korea	25	27583868	Cho et al.,	2016	Prospective cohort	3.98 years	412	MetS: 66	MetS: 16.0%; 40	30.6	23.5	NDGG	ADA 2010
	Thailand	39	29926712	Ruksasakul et al.,	2016	case control	2.97 years	56	MetS: 15	26.8%; 90	38.6 (follow-up)	24.6	CC	AHA/NHLBI criteria
	Iran	58	25892996	Valizadeh et al.,	2015	Prospective cohort	22.8 months (1,9 years)	110	MetS: 22	20%; 105	>34:64.5% ≤34:35.5%	28.5	Did not define	Israelite National Committee Guidelines
Cardiovas-cular (CV) events	Israel	71	23749791	Kessous et al.,	2013	Prospective cohort	10 years	4928	Simple CV events (not specified): 365	Simple CV events: 7.4%; 741	32.4	not specified	NDGG	ICD
Non-Alcoholic Fatty Livery Disease (NAFLD)	India	72	32961610	Kubihal et al.,	2021	Cross-sectional	16 months (9-38 months)	201	NAFLD: 126	NAFLD: 62.7%; 63	31.9	26.3	IADPSG	Fibroscan

N.A., Not available; T2D, type 2 diabetes; HTN, hypertension; MetS, metabolic syndrome; GDM, gestational diabetes mellitus; BMI, body mass index; AHA, American Heart Association; NHLBI, National Heart Lung and Blood Institutes; ICD, International Classification of Diseases; IDF, International Diabetes Federation; NCEP ATPIII, National Cholesterol Education Program Adult Treatment Panel III; ESH-ESCG, European Society of Hypertension-European Society of Cardiology Guidelines; MHS, Maccabi Healthcare Services; JSOG, Japan Society of Obstetrics and Gynecology; CC, Carpenter-Coustan; ADA, American Diabetes Association; WHO, World Health Organization; NDDG, National Diabetes Data Group; IADPSG, International Association of Diabetes and Pregnancy Study Groups; MOH, Ministry of Health.

**Criteria of Abell and Beischer:** GDM was defined as if 3hr 50g OGTT of any 2 abnormal glucose readings: 0-hr ≥5.0 mmol/L; 1-hr ≥9.5 mmol/L; 2-hr ≥8.1 mmol/L; 3-hr ≥ 7.0 mmol/L.

**Korean guidelines:** GDM was defined as if 3hr100g OGTT of any 2 abnormal glucose readings: 0-hr ≥ 5.8 mmol/L; 1-hr ≥10.6 mmol/L; 2-hr ≥ 9.2 mmol/L; 3-hr ≥ 8.1 mmol/L.

**Israelite National Committee Guidelines:** MetS was defined as having any three of the following traits: waist circumference > 95 cm in females; triglyceride ≥ 150 mg/dL (> 1.70 mmol/L) or drug consumption for elevated triglyceride levels; high-density lipoprotein < 50 mg/dL (< 1.30 mmol/L); systolic blood pressure ≥ 130 and/ or diastolic blood pressure ≥ 85 mm Hg or receiving antihypertensive drugs; and fasting plasma glucose ≥ 100 mg/dL (≥ 5.55 mmol/L) or consuming antiglycemic agents.

**IDF:** MetS was defined if had central obesity (waist circumference ≥90 cm in men or ≥80 cm in women) plus at least two of the following: (1) raised triglycerides >150 mg/dL (1.7 mmol/ L) or using specific treatment for this lipid abnormality; (2) reduced high-density lipoprotein cholesterol <40 mg/ dL (1.03 mmol/L) in men or <50 mg/dL (1.29 mmol/L) in women or using specific treatment for this lipid abnormality; (3) raised blood pressure (systolic ≥130 mmHg or diastolic ≥85 mmHg or using antihypertensive drugs); and (4) raised fasting plasma glucose >100 mg/dL (5.6 mmol/L) or previously diagnosed type 2 diabetes.

**NCEP ATPIII criteria:** MetS was defined if had at least three of the following: (1) waist circumference ≥90 cm in men, or ≥80 cm in women; (2) systolic blood pressure ≥130 mmHg, and/or diastolic blood pressure ≥85 mmHg, or using antihypertensive drug treatment; (3) fasting glucose ≥100 mg/dL, or using drug treatment for elevated glucose; (4) triglyceride ≥150 mg/dL or using drug treatment for elevated triglycerides; (5) high-density lipoprotein cholesterol <50 mg/dL in women, or <40 mg/ dL in men, or using drug treatment for reduced high-density lipoprotein cholesterol.

**AHA/NHLBI criteria:** MetS was defined if 3 out of the following 5 criteria are met, (1) waist circumference>80 cm, (2) blood pressure >130/85 mmHg or on antihypertensive medication, (3) fasting plasma glucose >100 mg/dL or on anti-diabetic medication, (4) fasting triglyceride >150 mg/dL, (5) high-density lipoprotein <50 mg/dL or on antihyperlipidemic medications.

**2007 ESH- ESC Guidelines:** hypertension was defined as systolic blood pressure ≥ 140mmHg or diastolic blood pressure ≥ 90 mmHg or taking antihypertensive medicines

*Incidence rate in per 100 000 person year is only calculated when the mean year of follow-up is above 1 year.

#### Prediabetes and T2D

It is well-known that women with a history of GDM have a substantially increased risk of developing T2D than counterparts without such a history (8). A systematic review and meta-analysis on prospective studies with reasonable retention rates (mainly on European women) suggested that the conversion rate from GDM to T2D was seven folds increased among women GDM after index pregnancy, compared with those who had a normoglycaemic pregnancy (RR 7.43, 95% CI: 4.79-11.51) (8).

Sixty-three studies described the postpartum incidence rate of prediabetes and T2D among mothers diagnosed with GDM in Asia, with sample sizes ranging from 35 to 11 270 subjects, most of which defined prediabetes and T2D using the same guidelines [e.g., WHO 1999 (41) or ADA 2014 guidelines (42)] even though their GDM diagnostic criteria differed. We reported the percentage incidence (%) if prediabetes or T2D was recorded within one year from delivery (mostly between 6 and 12 weeks). Then we reported person-years incidence (per 1000 person-years) if prediabetes or T2D was recorded beyond one year from delivery (up to 15 years).

Within one year from delivery, the conversion rate varied significantly between studies from GDM to prediabetes (11.9%-49.1%) and from GDM to T2D (1.1%-66.7%), respectively. Beyond one year after delivery, the incidence rate from GDM to T2D was the highest in South Asia (47 – 271 per 1000 person-years), followed by East Asia (9 – 110 per 1000 person-years). We noted inconsistencies with study estimates within the same region. For instance, one study in Iran reported a much higher incidence T2D conversion rate than another study in Iran (172 vs. 9 per 1000 person-years) (35, 43). Potential reasons for inconsistencies in the conversion rates from GDM to T2D could be the variation in studied population characteristics, duration of follow-up, retention rate, and data collection quality.

As for Asian immigrants, we identified only two reports comparing Asian immigrants with non-Asian counterparts, one from Spain with one-year follow-up (44) and the other from the US with an average 7.6-year follow-up (45). Both studies suggested that prediabetes and T2D conversion rates were higher in South Asian migrants than native NHW [prediabetes: 43.3% vs. 28.5% (44); T2D: 55 vs. 40 per 1000 person-years (45)].

Existing data on risk factors of T2D among women with a history of GDM were firstly reported in the NHW population, such as greater pre-pregnancy BMI (8, 9), excessive weight gain (3), unhealthy dietary patterns (3), physical inactivity (3), and a short period of lactation (3, 10). In the Asian population, there are also quite a few at-risk pre-natal maternal characteristics recently added to this pond of evidence, such as family history of diabetes (43), a higher degree of consanguineous marraiges (43), higher pre-pregnancy BMI (29, 31, 32, 46), higher total cholesterol quartile at GDM diagnosis during the index pregnancy (47), younger age at delivery (<30 years) (46), and a short period of lactation (<6 months) (33). Post-natal risk such as missing medical assistance in the continuum of GDM care after delivery could be another risk for T2D progression among Asian mothers with a history of GDM (48).

#### Cardiovascular Disorders

##### Hypertension

A history of GDM was related to increased risk of hypertension (HTN) after the index pregnancy in some but not all studies. For instance, the US Nurses’ Health Study found an increased risk of postpartum HTN among women with a history of GDM (49). In contrast, a Dutch cohort suggested the risk of developing HTN was mainly significant among women with a history of hypertensive disorders during pregnancy (HDP) rather than GDM (50). Among the three studies identified in our review on GDM and subsequent hypertension risk (28, 30, 38), the Chinese Tianjin GDM prevention program reported a much higher incidence rate of HTN among women diagnosed with HDP and GDM than women with GDM alone (118 vs. 26 per 1000 person-years) (38), which partially agreed with the Dutch cohort.

The mechanisms underlying postpartum HTN in women with GDM remain un-elucidated. Insulin resistance may be a component of the underlying pathophysiology linking GDM with postpartum HTN, with or without HDP (51). As we know, obesity and excessive weight gain during pregnancy are associated with insulin resistance (38), inflammation and oxidation (52), all of which may lead to permanent vascular damage (51) and even irreversible peripheral vascular resistance. Due to the largely inadequate evidence, future research to investigate the role of antenatal and postpartum lifestyle (e.g., dietary patterns, physical activities) in the progression of HTN is warranted in Asians.

##### Cardiovascular Risks and Cardiovascular Diseases

Emerging evidence has led to the increasing recognition of the association between GDM and cardiovascular (CV) risks and CV events later in life (53). Previous studies in the Western population have identified a higher level of inflammatory (e.g., C-reactive protein) (54), vascular endothelial dysfunction (e.g., intimal medial thickness) (55), and a 2-7 times higher risk of coronary artery calcification or CVD after 12-15 years’ follow-up (56–58), among women with a history of GDM. In Asia, five studies reported metabolic syndrome in Asian women with a history of GDM, with an incidence rate ranging from 40 to 90 per 1000 person-years. One Chinese study reported postpartum dyslipidemia (38.5%) among women with a history of GDM (47), while the other Israelite study reported a 30-70% higher risk of developing CV events and CV hospitalization among women with a history of GDM, even after adjusting for pre-eclampsia and maternal obesity at index pregnance (39).

Thus far, only determinants for postpartum CVD risks and CV events were reported as family history of T2D (59) and postpartum development of T2D (58) in the western population. Even though postpartum CVD determinants among women with GDM have yet to be fully investigated, long-standing exposure to cardio-metabolic risks has been speculated in the GDM-CVD link.

#### Cancer

GDM was associated with 30-40% increased risks of breast cancer, thyroid cancer, stomach cancer, and liver cancer for all races and ethnicities in a recent meta-analysis (60). As in the Asian population alone, we identified six retrospective cohort studies (Taiwan, South Korea and Israel) using either national insurance or a medical database to investigate the association between GDM and various cancers. All of them reported higher incidences of breast cancer, thyroid cancer, pancreatic cancer, ovarian cancer, lung cancer, and kidney cancer among the Asian female population with a history of GDM after a median of 5-38 years of follow-up than those parous women without such a history. For example, the incidence rate of cancer among Israelite women with a history of GDM was reported in breast (2 per 1000 person year) (37) and ovary (1 per 100 person year) (36), respectively.

It has been well documented that T2D is associated with higher risks of all-cancer incidence (61), especially malignancies in the breast, pancreas, and liver in women (62, 63). Some evidence has alluded to the mitogenic effect while binding to the insulin-like growth factor-I receptor secondary to insulin resistance (64). Furthermore, hyperglycemia itself might promote carcinogenesis *via* increasing oxidative stress (65, 66). However, data regarding cancer risks associated with GDM are merely gathered in the Western population.

#### Liver Dysfunction

Liver dysfunction is a common cause of chronic liver disease that affects approximately one in four adults worldwide, which is characterized by liver steatosis (fat deposition), inflammation, and hepatocyte damage (67). Researchers have suggested a link between metabolic risks (i.e., obesity, hyperglycemia, hyperlipidemia, and insulin resistance) and hepatic fatty deposition and non-alcoholic fatty liver disease (NAFLD) in the past decades (68, 69). Notably, women with a history of GDM were found to have raised liver triglyceride (TG) levels, highlighting a potential link between GDM and liver dysfunction (70, 71). Despite the higher prevalence of postpartum liver fat (72), abnormal liver score (73) and even NAFLD (71, 74), such results were mostly gathered from the Western population. There is one study from South Asia (India) reported a 2.11-fold higher odds of NAFLD among women with GDM, compared with women without GDM. The researchers suggested that postpartum medical conditions such as overweight/obesity, metabolic syndrome, and prediabetes were risk factors for developing NAFLD, during a median of 16 months’ follow-up after delivery (40).

### Adverse Health Outcomes of Offspring Born From Pregnancies Complicated by GDM

#### Overview

A body of evidence has implied that specific developmental programming in offspring is influenced by maternal hyperglycemia; in particular, epigenetic modification may be the key underlying mechanism (75, 76). Our review identified forty-two studies conducted on Native Asians (**Table 2**) and eight studies conducted on Asian immigrants (**Supplementary Table 7**) with up to 18 years’ follow-up, all of which were within the research scope of adverse health outcomes among offspring born to mothers with GDM. Offspring health outcomes, including fetal growth and neonatal anthropometric measures, were reported in Native Asians and Asian migrants, whereas offspring health outcomes, including congenital anomalies, neuro-cognitive function, and cardio-metabolic phenotypes, were only reported in Native Asians (**Figure 4**). None of these studies investigated risk factors underlying maternal GDM and the development of offspring health outcomes. Among 50 included studies in this topic, fourteen (28%) were assessed low in risk of bias, while the rest 72% were assessed either high or very high in risk of bias.

**Table 2:** Summary of GDM-related offspring health outcomes in Asians.

Offspring outcomes	Country	No	PMID or Doi	Author	Year	Study design	Mean or range of follow-up	Total offspring number and outcomes definition	Baseline maternal age,&offspring age	Multiple variable adjustment	Effect size (referencing to non-GDM mothers)	
**Fetal outcomes**												
**Athropo-metry**	India	1	27913848	Venkataraman et al.	2016	Prospective cohort	during pregnancy	153 fetus with GDM mothers, 178 fetus with non-GDM mothers	Mom: 28.6 years Fetus: 20 wks GA; 28-32 wks GA	Maternal age, BMI, parity, gestational weight gain, fetal sex and gestational age	Fetus born to GDM mothers had significantly thicker anterior abdominal wall thickness (20 weeks: 0.26 mm, 0.15-0.37, p<0.0001; 28-32 weeks: 0.48, 0.30-0.65, p<0.0001).	
**Neonatal outcomes**												
**1. Anthropometry**	China	2	33407256	Hu et al.	2021	Prospective cohort	at birth	205 newborns born to GDM mothers 740 newborns born to non-GDM mothers	Mom: 31.3 years Offspring: newborn	Age of infants at each measurement, pre-pregnancy BMI, maternal age, parity and gestational age	Offspring born to mothers with GDM had higher weight-for-length z-score (WFLZ) [β: 0.26 SD units (95% CI: 0.13–0.40)] across infancy than those of mothers without GDM.	
		3	29886780	Yan et al.	2020	Prospective cohort	at birth	Macrosomia: n=630 born to GDM mothers (n=8272); n=2121 for born to non-GDM mothers (n=34085)	Mom: 30.5 years Offspring: newborn	Crude model	Infants born to GDM mothers had lower macrosomia rate (1.5%) while infants born to non-GDM mothers had higher macrosomia rate (4.9%).	
		4	31731641	Cheng et al.	2019	Prospective cohort	at birth	Macrosomia: n=13 born to GDM mothers (n=97); n=51 born to non-GDM mothers (n=853)	Mom: did not mention Offspring: newborn	Maternal age, education, average monthly household income, postpartum BMI, parity, passive smoking, family history of diabetes, iron supplementation, multivitamin supplementation, gestational dietary intake, and alcohol use.	Infants born to GDM mother had higher risk of macrosomia (RR: 2.11, 95% CI: 1.16-3.83).	
		5	31271809	Yang et al.	2019	Prospective cohort	at birth	Macrosomia: n=238 born to GDM mothers (n=1495); n=1553 born to non-GDM mothers (n=18127). LGA: n=240 for GDM mothers (n=1495); n=1486 born to non-GDM mothers (n=18127).	Mom: 28.5 years Offspring: newborn	Maternal age, family history of diabetes, height, parity, nationality, GA at delivery, child gender, smoking or alcohol use before or during pregnancy, intervention for GDM.	Infants born to GDM mothers had higher risk of having macrosomia (OR: 2.70, 95% CI: 2.15-3.40) and LGA (OR: 2.57, 95% CI: 2.05-3.21).	
		6	30412096	Ding et al.	2018	Retrospective cohort study	at birth	Macrosomia: n=178 born to GDM mothers (n=3221)	Mom: 32.7 years Offspring: newborn	Crude model	Based on the OGTT results, women had three abnormal glucose values had more macrosomia (46/406; 11.3%) than women had two (51/939; 5.4%) or one (81/1876; 4.3%) abnormal glucose values (p<0.001).	
		7	27806670	Wang et al.	2017	Retrospective cohort study	at birth	Macrosomia: n=447 born to GDM mothers (n=3683); n=7875 born to non-GDM mothers (n=123906)	Mom: did not mention Offspring: newborn	Crude model	Infants born to GDM mothers had an increased risk of macrosomia (OR: 2.42; 95% CI: 2.26-2.59).	
		8	26496961	Zhao et al.	2015	Prospective cohort	5-10 years	LGA: n=150 born to GDM mothers (n=1068); n=183 born to non-GDM mothers (n=1756)	Mom: 29.8 years Offspring: newborn	Crude model	GDM mothers had higher rate of LGA infants (14% vs. 10.4%, p=0.005), compared with non-GDM mothers.	
		9	26401753	Wang et al.	2015	Prospective cohort	at birth	Macrosomia: n=49 born to GDM mothers (n=587: 114 obese vs. 473 non-obese); n=33 born to non-GDM mothers (n=478). LGA: n=182 born to GDM mothers (n=587: 114 obese vs. 473 non-obese); n=136 born to non-GDM mothers (n=478)	Mom: 30.2 years Offspring: newborn	Maternal age and gestational weeks.	No difference in macrosomia and LGA between infants born to GDM and non-GDM mothers. Infants born to obese GDM mothers had higher macrosomia (p=0.001) and LGA (p<0.001) prevalence than non-obese GDM mothers.	
		10	26376766	Chen et al.	2015	Prospective cohort	at birth	LGA: n=97 born to GDM mothers (n=1049)	Mom: 29 years Offspring: newborn	Crude model	Compared with normal weight GDM mothers, Infants born to overweight or obese GDM mothers had higher risk of LGA than normal weight GDM mothers (OW: OR 3.8; 95% CI: 2.0–7.0; OB: OR 2.0; 95% CI: 1.2–3.3). Compared with normal GWG mothers with GDM, infants born to GDM mothers with excessive GWG had higher risk of LGA (OR: 3.3; 95% CI: 2.1–5.1).	
	Bangladesh	11	http://doi.org/10.3329/jom.v13i2.12749	Mannan et al.	2012	Cross-sectional study	at birth	Macrosomia: n=10 born to GDM mothers (n=72); n=2 born to non-GDM mothers (n=72).	Mom: 15-25 yrs: 69.5% 26-35 yrs: 23.6% 36-45 yrs: 6.9% Offspring: newborn	Crude model	Newborn born to mother prior to GDM had a higher macrosomia prevalence (13.9% vs. 2.8), compared with those born to non-GDM mothers.	
	South Korea	12	9314639	Jang et al.	1997	Case-control study	at birth	Macrosomia: n=9 born to GDM mothers (n=65); n=5 born to non-GDM mothers (n=153) LGA: n=26 born to GDM mothers (n=65); n=20 born to non-GDM mothers (n=153)	Mom: 31.3 years Offspring: newborn	Crude model	Infants born to GDM mothers had significantly higher rates of macrosomia (13.8% vs. 3.3%) and LGA (40% vs. 13.1%), compared with non-GDM mothers.	
	Kuwait	13	30944829	Groof et al.	2019	Cross-sectional study	at birth	Macrosomia: n=16 born to GDM mothers (n=109); n=43 born to non-GDM mothers (n=758)	Mom: <25 yrs: 16.6% 25-29 yrs: 30.0% 30-34 yrs: 29.4% ≥35 yrs: 24.0% Offspring: newborn	Maternal nationality, pre-pregnancy BMI, and family history of GDM	Infants born to GDM mothers had a higher risk of macrosomia (OR = 2.36; 95% CI: 1.14, 4.89).	
	Israel	14	33236556	Riskin et al.	2020	Retrospective cohort study	At birth	LGA: n=50 born to GDM mothers (n=479); n=34 born to non-GDM mothers (n=526).	Mean: 33.0 years	Crude model	10.4% of newborns born to GDM mothers had LGA while 6.5% of newborns born to non-GDM mothers had LGA (p<0.001).	
		15	29429374	Walter et al.	2019	Retrospective cohort study	18 years	Macrosomia: n=1318 born to GDM mothers (n=11999); n=9957 born to non-GDM mothers (n=118623)	Mom: 30.5 years Offspring: 18 years	Crude model	Infants born to GDM mothers had higher rates of macrosomia (11.0%).	
**2. Birth condition**	Israel	14	33236556	Riskin et al.	2020	Retrospective cohort study	At birth	Hypoglycemia: n=34 born to GDM mothers (n=479); n=9 born to non-GDM mothers (n=526). Polycythemia: n=180 born to GDM mothers (n=479); n=33 born to non-GDM mothers (n=526). Hypertrophic cardiomyopathy: n=7 born to GDM mothers; none from the non-GDM mothers (n=526).	Mean: 33.0 years	Crude model	Compared with newborn born to non-GDM mothers, newborn born to GDM mothers had 3.6 odds of hypoglycaemia and 11.1 odds of polycythemia at birth.	
	Malaysia	16	31778255	Samsuddin et al.	2020	Prospective cohort	at birth	Hypoglycaemia: n=11 born to GDM mothers (n=145); n=7 born to non-GDM mothers (n=362).	Mom: 32.3 years Offspring: newborn	Crude model	Infants born to GDM mothers had higher rate of hypoglycaemia (9.2% vs. 1.9%), compared with non-GDM mothers.	
	Saudi Arabia	17	26409797	Alfadhli et al.	2015	Prospective cohort	at birth	Apgar score <7 at 5 minutes: n=23 born to GDM mothers (n=292); n=3 born to non-GDM mothers (n=281) Hypoglycaemia: n= 40 born to GDM mothers (n=292); n=4 born to non-GDM mothers (n=281).	Mom: 32.3 years Offspring: newborn	Crude model	Infants born to GDM mothers had higher risk of neonatal low Apgar score (OR: 5.55; 95% CI: 1.58-19.48) and hypoglycaemia (OR: 9.35; 95%CI: 2.79-31.25).	
	Thailand	18	26111427	Luengmetta-kul et al.	2015	Retrospective cohort study	at birth	Hypoglycaemia: n=25 born to GDM mothers (n=487); n=2 born to non-GDM mothers (n=345). Hyperbilirubinemia: n=67 born to GDM mothers (n=487); n=27 born to non-GDM mothers (n=345).	Mom: 32.6 years Offspring: newborn	Crude model	Infants born to GDM mothers had a higher risk of hypoglycaemia (OR: 12.3; P < 0.0001) and neonatal hyperbilirubinemia (OR, 1.9; P = 0.013).	
	Thailand	19	24372900	Youngwani-chsetha et al.	2013	Prospective cohort	at birth	Hypoglycaemia: n=50 born to GDM mothers (n=118).	Mom: 32.6 years Offspring: newborn	Crude model	The incidence of neonatal hypoglycaemia was 42.4% among women with a history of GDM	
	India	20	24944938	Mahalakshmi et al.	2014	Retrospective study	at birth	Hypoglycaemia: n=22 born to GDM mothers (n=174).	Mom: 29 years Offspring: newborn	Crude model	The incidence of neonatal hypoglycaemia was 12.6% among women with a history of GDM	
	Bangladesh	11	http://doi.org/10.3329/jom.v13i2.12749	Mannan et al.	2012	Cross-sectional study	at birth	Hyperbilirubinemia: n=60 born to GDM mothers (n=72); n=6 born to non-GDM mothers (n=72). Respiratory distress syndrome: n=8 born to GDM mothers (n=72); n=3 born to non-GDM mothers (n=72).	Mom: 15-25 yrs: 69.5% 26-35 yrs: 23.6% 36-45 yrs: 6.9% Offspring: newborn	Crude model	More babies also suffered from neonatal jaundice (22.2% vs 8.4%, p<0.05) and respiratory distress syndrome (11.1% vs 4.17%, p<0.05) in GDM groups than non-GDM groups.	
	Turkey	21	322558417	Vijay et al.	2020	Case-control	At birth	Vitamin D deficiency (serum values < 20ng/ml): 30 infants born to GDM mothers (n=30); 13 infants born to non-GDM mothers (n=30)	Mom: 30 years old.	Crude model	The mean value of Vitamin D levels in GDM babies was 8.47ng/ml and was 19.51ng/ml in the control (p value <0.001).	
**3. Congenital anomalies**	China	6	30412096	Ding et al.	2018	Retrospective cohort study	at birth	Fetal malformations (did not specify): n=33 born to GDM mothers (n=3221)	Mom: 32.7 years Offspring: newborn	Crude model	Female malformation rate born to GDM mothers was 1.02%.	
	Turkey	22	DOI:10.5262/tndt.2017.1002.05	Soylu et al.	2017	Case–control study	0-18 years	21 born to GDM mothers, 259 born to non- GDM mothers CAKUT: n=14 for GDM newborns; n=126 for non- GDM newborns	Mom: Did not mention Offspring: CAKUT cases: 6.9 years, Non-CAKUT controls: 5.6 years	Crude model	CAKUT had 10% children born to GDM mothers and the controls only had 5% children born to GDM mothers. However, it is not statistically significant.	
	Taiwan	23	26844492	Tain et al.	2016	Case–control study	at birth	10543 born to GDM mothers, 1591179 born to non-GDM mothers. Among them: Congenital anomalies of kidney and urinary tract (CAKUT) : n=11 born to GDM mothers; n=0 born to non-GDM mothers; Musculoskeletal system anomalies: n=33 born to GDM mothers; n=2753 born to non-GDM mothers; Eye and face anomalies: n=29 born to GDM mothers; n=2626 born to non-GDM mothers; Heart and circulatory system anomalies: n=28 born to GDM mothers; n=1623 born to non-GDM mothers; Genitourinary system: n=20born to GDM mothers; n=1188 born to non-GDM mothers.	Mom: did not mention Offspring: newborn	Crude model	Infants born to GDM mothers had higher risks of CAKUT (OR 2.22; 95% CI: 1.06-4.67), and also higher prevalence of musculoskeletal system (0.32% vs. 0.17%, p<0.001), eye and face (0.28% vs. 0.17%, p<0.001), heart and circulatory system (0.27% vs. 0.10%, p<0.001) and genitourinary system (0.19% vs. 0.07%, p<0.001), compared those born to non-GDM mothers.	
	China	24	26071138	Liu et al.	2015	Retrospective cohort study	6 months	Congenital heart disease: n=206 born to GDM mothers (n=3060); n=17371 born to non-GDM mothers (n=87736).	Mom: did not mention Offspring: 6 months	Crude model	Male infants born to GDM mothers had increased risk of congenital heart disease (OR 2.56; 95% CI: 1.71-3.83).	
	India	20	24944938	Mahalakshmi et al.	2014	Retrospective cohort study	at birth	Congenital anomalies (did not specify): n=9 born to GDM mothers (n=174)	Mom: 29 years Offspring: newborn	Crude model	Congenital anomalies was 5.2% in GDM mothers.	
	Bangladesh	11	http://doi.org/10.3329/jom.v13i2.12749	Mannan et al.	2012	Cross-sectional study	at birth	Congenital malformation (did not specify): n=1 born to GDM mothers (n=72); n=2 born to non-GDM mothers	Mom: 15-25 yrs: 69.5% 26-35 yrs: 23.6% 36-45 yrs: 6.9% Offspring: newborn	Crude model	There is no difference between GDM group and non-GDM group regarding congenital malformation.	
**4. Neuro-Cognitive Structure and Function**	China	25	33196602	Xuan et al.,	2020	Case-control	First 33-day after delivery	31 infants with corrected GA at delivery (33.42-36.00 weeks) born to GDM mother; and 31 GA and sex-matched infants born to non-GDM mothers	31.5 years Offspring: first 33 days postpartum	Crude model	Fractional anisotropy was significantly decreased in the splenium of corpus callosum, posterior limb of internal capsule, thalamus in infants born to GDM mothers, reflecting microstructural white matter abnormalities in the GDM group.	
**Child outcomes**												
**1. Anthro-pometry, Blood pressure, and CV risks**	China	26	33633685	Du et al.,	2021	Prospective cohort	1 year old	389 infants born to GDM mothers; 778 infants born to non-GDM mothers matched with offspring gender.	Mom: 32.1 years Offspring: 1 year old	Maternal age, family history of diabetes, parity, gestational weight gain, pre-pregnancy BMI, maternal gestational hypertension, GA, birth weight, birth length, mode of delivery, parental smoking, breastfeeding status, weaning months.	Maternal GDM was found to be independently and significantly associated with overweight or obesity in 1-year aged female offspring only (OR 1.61, 95% CI 1.09–2.37, p < 0.05).	
		27	32861332	Liang et al,	2020	Case-control	6 years old	560 infants born to GDM mothers; 554 infants born to non-GDM mothers matched with age and sex-frequency	Mom: 30.0 years Offspring: 6 years old	Maternal age at pregnancy, gestational weight gain, gestational age at delivery, numbers of childbirth, smoking status, drinking status, marital status, education, gestational hypertension, occupation of mothers, family history of diabetes, family monthly income treatment of GDM and maternal pre-pregnancy BMI.	There is an interaction between maternal BMI genetic risk score (GRS) and GDM status in relation to childhood overweight or obesity. Per unit of GRS was associated with a 24% (*P*<.001) and a 28% (*P*<.001) increased risk of overweight and obesity among children of GDM mothers, whereas no significant associations were observed among children of mothers without GDM.	
		28	30181654	Wang et al.	2019	Prospective cohort	1-6 years old	1500 born to GDM mothers, 25655 born to non-GDM mothers N.A.	Mom: 28.5 years Offspring: each year measured from year 1-year 6	Maternal age and ppBMI, education, smoking status, infant feeding and total GA.	Children born to GDM mothers had consistently greater BMI z-score and risk of overweight from year 1 to year 6.	
		29	28120866	Zhang et al.	2017	Cross-sectional study	1-5 years	1263 born to GDM mothers Childhood obesity: n=128 Childhood central obesity: n=126 Childhood hyperglycemia: n=126	Mom: 30 years Offspring: each year from year 1 to year 5	N.A.	N.A.	
		8	26496961	Zhao et al.	2015	Prospective cohort	5-10 years	Childhood overweight: n=177 born to GDM mothers (n=1068); n=221 born to non-GDM mothers (n=1756). Childhood obesity: n=114 born to GDM mothers (n=1068); n=210 born to non-GDM mothers (n=1756).	Mom: 29.8 years Offspring: Year 1-10	Maternal ppBMI, child gender, total GA, infant feeding.	At age 1–2 and 2–5 years, no difference in overweight (11.0 v. 12.0%, P=0.917, and 15.7 v. 14.6%, P=0.693, respectively) between children born to GDM and non-GDM mothers. At age 5–10 years, children born to GDM mothers had higher risk of being overweight and obesity (OR: 2.28, 95% CI 1.61–3.22).	
		30	25716565	Chang et al.	2015	Retrospective cohort study	6 years	356 born to GDM mothers, 500 born to non-GDM mothers.	Mom: 28.6 years Offspring: 6 years	Crude model		Children born to GDM mothers had higher BMI (15.8 vs. 12.3, p=0.001), higher sum of skinfold (Subscapular skinfold thickness + Triceps skinfold thickness) (8.2 vs. 4.8cm, p=0.03), compared with those born to non-GDM mothers.
		31	24689042	Liu et al.	2014	Prospective cohort	at 1 year	1420 born to GDM mothers, 25737 born to non-GDM mothers.	Mom: 29.2 years Offspring: birth, 3 months, 6 months, 9 months, 12 months	Crude model		Infants born to GDM mothers had bigger change in mean values of z-scores for birth length-for-gestational age (0.16 vs. -0.08), birth weight-for-length (0.30 vs. -0.001), from birth to month 3, and bigger changes in mean value in z-scores from month 9-12 (0.05 vs. 0.02), compared with infants born to non-GDM mothers.
		32	22160003	Andegiorgish et al.	2012	Cross-sectional study	N.A.	Childhood overweight: n=15 born to GDM mothers (n=24); n=518 born to non-GDM mothers (n=1527).	Mom: Did not mention Offspring: 7-11 years & 12-18 years	Paternal obesity and maternal obesity.		Children born to GDM mothers had higher rate of overweight (2.8% vs. 0.9%, p=0.003), compared with those born to non-GDM mothers. Children born to GDM mother had a higher risk of overweight (OR: 2.76; 95% CI: 1.11–6.87).
	Hong Kong	33	29777227	Hui et al.	2018	Prospective cohort	Month 3-year 16	539 born to GDM mothers, 6758 born to non-GDM mothers N.A.	Mom: ≤24 yrs: 7.3% 25-29 yrs: 27% 30-34 yrs: 40% ≥35 yrs: 26% Offspring: 3 and 9 months; 2–8 years; 8–16 years	Maternal age and birth place, SES, parental education, presence of PE, maternal smoking and BMI at visit, history of T2D, Child sex, parity and age at visit.		Children born to GDM mothers had a lower BMI z-score during infancy (-0.13, 95% confidence interval (CI) -0.22, -0.05) but higher BMI z-scores during childhood (0.14, 95% CI 0.03, 0.25) and adolescence (0.25 95% CI 0.11, 0.38). Breastfeeding for the first three months did not modify the association.
		34	28279981	Tam et al.	2017	Prospective cohort	7 years	Childhood overweight or obesity (BMI>=85th percentile): n=30 born to GDM mothers (n=123), n=121 born to non-GDM mothers (n=803). Prediabetes: n=5 born to GDM mothers; n=13 born to non-GDM mothers. T2D: n=1 born to GDM mothers; n=0 born to non-GDM mothers.	Mom: Did not mention Offspring: 6.9 years	Crude model		Offspring born to GDM mothers had higher rates of abnormal glucose tolerance (4.7%vs. 1.7%; P = 0.04), higher rates of overweight or obesity, greater BMI, higher blood pressure, lower oral disposition index, and a trend toward reduced b-cell function, compared with those born to mothers without GDM.
		35	19047239	Tam et al.	2008	Prospective cohort	8 years	63 born to GDM mothers, 101 born to non-GDM mothers	Mom: 28.5 years Offspring: 7.7 years	Child age and gender.		Children born to GDM mothers had higher SBP (94 vs 88 mm Hg) and DBP (62 vs 57 mm Hg) and lower HDL (1.58 vs 1.71 mmol/L) levels, compared with those born to non-GDM mothers.
	India	36	25478935	Krishnaveni et al.	2015	Prospective cohort	13.5 years	26 born to GDM mothers, 208 born to non-GDM mothers	Mom: Did not mention Offspring: 13.5 years	Child age, sex, socioeconomic status, and children’s current weight.		Children born to GDM mothers had higher insulin level (54.3 vs. 42.5 pmol/L, p=0.02), higher SBP (mean difference: 5.96; 2.10-9.82) and higher insulin resistance (2.0 vs. 1.6, p=0.02) than those born to non-GDM mothers. Children born to GDM mothers had higher cardia output (0.49, 0.26-0.72), stroke volume 3.98 (2.00, 5.97) and lower total peripheral resistance (-114; -220~-9), compared with those born to non-GDM mothers.
		37	19918007	Krishnaveni et al.	2010	Retrospective cohort study	9.5 years	35 born to GDM mothers, 420 born to non-GDM mothers.	Mom: Did not mention Offspring: 9.5 years	Crude model		Children born to GDM mothers had more adiposity and higher SBP and insulin resistance, compared with control children at age 5 years. And such effects were greater at age 9.5 years.
	Israel	38	21804818	Tsadok et al.	2011	Prospective cohort	17 years	293 born to GDM mothers, 59499 born to non-GDM mothers	Mom: 31.2 years Offspring: 17 years	Birthweight		GDM remained significantly associated with offspring 17-year BMI (1.17; 0.81, 1.52) and diastolic BP (1.52; 0.56, 2.48).
	Sri Lanka	39	32670637	Herath et al.	2020	Retrospective cohort study	10 years	Overweight: n= 49 born to GDM mothers (n=159); n=41 born to non-GDM mothers (n=253). Abdominal obesity: n=24 born to GDM mothers (n=159); n=6 born to non-GDM mothers (n=253).	Mom: 31.9 years Offspring: 10.9 years	Maternal BMI, maternal age at delivery, and birth order.		Children born to GDM mothers had higher median BMI (17.6 vs 16.1, p< 0.001), waist circumference (63 cm vs 59.3 cm, p< 0.001), and triceps skinfold thickness (13.7mm vs 11.2 mm, p< 0.001), and also higher risk of overweight (OR: 2.6, 95% CI 1.4–4.9) and abdominal obesity (OR:2.7, 95% CI 1.1–6.5) at the age of 10-11 years.
	Pakistan	40	30940265	Hoodbhoy et al.	2018	Retrospective cohort study	2-5 years	53 born to GDM mothers, 83 born to non-GDM mothers	Mom: 30.8 Offspring: 2-5 years	Crude model		Children born to GDM mothers with medication had a decreased mitral E/A ratio [IQR] = 1.7 [1.6-1.9] and 1.56 [1.4-1.7], respectively, p = 0.02), compared with those born to GDM mothers treated by diet only, and also a higher cIMT (0.48 vs. 0.46, p = 0.03), compared with those born to non-GDM mothers. There was no significant difference in offspring cardiac morphology, myocardial systolic and diastolic function, and macrovascular assessment GDM and non-GDM groups.
**2.Cognitive function**	India	41	20614102	Veena et al.	2010	Prospective cohort	9.7 years	32 born to GDM mothers, 483 born to non-GDM mothers	Mom: 26.0 years Offspring: 9.7 years	Child’s age, sex, gestation, neonatal weight and head circumference, maternal age, parity, BMI, parent’s socio-economic status, education and rural/urban residence.		Children born to GDM mothers had significant higher learning, long-term retrieval/storage (β: 0.4SD, 95% CI: 0.01-0.75; p=0.042) and better verbal ability (0.5SD, 0.09-0.83; p=0.015).
**3.Endocri-nological and Ophthamo-logical morbidity**	Israel	15	29429374	Walter et al.	2019	Retrospective cohort study	18 years	11999 born to GDM mothers, 226623 born to non-GDM mothers Ophthalmic nfection/inflammation: n=89 born to GDM mothers (n=11999); n=1359 born to non-GDM mothers (n=226623).	30.5 18 years old	Crude model		Young adults born to GDM mothers treated by medication had higher risk of offspring ophthalmic related hospitalization (HR: 1.6, 95% CI: 1.1-2.4) compared with non GDM mothers.
	Israel	42	31117838	Shorer et al.	2019	Retrospective cohort study	18 years	9312 SGA infants: 259 born to GDM mothers, 9053 born to non-GDM mothers Among all SGA offspring: Thyroid disease: n=0 born to GDM mothers; n=8 born to non-GDM mothers. T1D and T2D: n=0 born to GDM mothers; n=7 born to non-GDM mothers. Hypoglycemia: n=1 born to GDM mothers; n=18 born to non-GDM mothers. Childhood obesity: n=1 born to GDM mothers; n=7 born to non-GDM mothers. Parathyroid hormone disease: n=0 born to GDM mothers; n=3 born to non-GDM mothers. Adrenal hormone disease: n=0 born to GDM mothers; n=2 born to non-GDM mothers.	Mom: 28.9 years Offspring: 18 years	Maternal hypertensive disorders, preterm birth, and maternal age		SGA children born to GDM mothers was not associated with higher risk of long-term endocrine morbidity of the offspring (adjusted HR 1.2, 95% confidence interval 0.27–5.00, p=0.82).

GDM, gestational diabetes mellitus; DM, diabetes mellitus; HC, head circumference; AC, abdomen circumference; FL, femur length; BPD, biparietal diameter; BMI, body mass index; LGA, large for gestational age; OR, odds ratios; OGTT, oral glucose tolerance test; CAKUT, congenital anomalies of the kidney and urinary tract; SD, standard deviation; HR, hazard ratio; BP, blood pressure; cIMT, carotid intima media thickness.

#### GDM and Fetal Growth

*In-utero* over nourishment can lead to fetal overgrowth, and such influence may predispose the offspring to obesity and T2D later in life if there is an obesogenic environment (84). A cohort in India reported an association between GDM and antenatal fetal growth at mid-late trimester (85). In this prospective cohort, fetuses of women with GDM had a thicker anterior abdominal wall while smaller femur length and biparietal diameter than fetuses of women without GDM. The researcher referred to this as “the thin-fat-phenotype” which represented a predisposition to T2D at birth (85).

Among Asian immigrants, one Norwegian study found that fetuses exposed to maternal GDM tended to be smaller in fetal weight at 24 weeks of gestation but thereafter grew faster until delivery, compared with fetuses not exposed to maternal with GDM (86). This trend was more prominent in South Asian women (86).

#### GDM and Neonatal Outcomes

##### Anthropometric Outcome At Birth

It is well-accepted that GDM is related to increased risk for macrosomia and large for gestational age (LGA) (6). We identified 14 papers that focused on this topic, with sample sizes ranging from 72 to 11 999 neonates. Among them, the majority reported consistent findings on either higher prevalence rates (11% to 40%) or higher risk ratios (2.0-2.7 times) of macrosomia or LGA among neonates born to GDM mothers, compared with their non-GDM counterparts, despite a couple reported otherwise. Interestingly, one study specifically looked at different combinations of glycemic abnormalities (fasting, 1-hour, and 2-hour glycemic levels) with macrosomia (77). The researchers found that women with three abnormal OGTT glycemic values had a much higher macrosomia rate in their offspring than those with two or one abnormal glycemic value (77). Such results—to some extent—suggested there might be remarkable neonatal outcomes specific to different GDM phenotypes (77).

Four studies reported neonatal birth size in Asian migrants equivocally. The US studies showed no differences in macrosomia rate between neonates born to NHW and Asian women with GDM (87, 88). In contrast, compared with the NHW counterparts, the Dutch study showed a lower macrosomia rate in offspring born to West Asian migrants (Turkish) (89) (18.6% vs. 22.6% [NHW]), while the Canadian study found that newborns born to South Asian female migrants had a greater skinfold thickness (11.7 vs 10.6 mm [NHW]; p=0.0001) (90).

##### Neonatal Health Ouctomes

Eight papers reporting other neonatal conditions were identified in our review, ranging from 72 to 10 543 in sample size. Neonatal disorders were listed as hypoglycemia, low Apgar score, hyperbilirubinemia/jaundice, polycythemia and respiratory distress syndrome. All studies consistently reported that neonates born to women with GDM were more susceptible to hypoglycemia, hyperbilirubinemia, respiratory distress syndrome and low Apgar score (<7 at 5 minutes), compared with those born to women without GDM.

##### Congenital Diseases

A total number of six studies reported findings on this topic, only half of which had specified the type of malformation as either congenital heart disease or congenital anomalies of the kidney and urinary tract (CAKUT). In general, evidence showed that neonates born to mothers with GDM tended to have a 2-3 times higher risk of developing congenital heart disease and CAKUT, especially more evident in male neonates (79). Despite the unclear pathophysiological mechanism, it has been speculated that serial maternal antenatal characteristics could affect embryonic development during the first trimester, such as pre-existing diabetes prior to pregnancy, overweight and obesity, and excessive weight gain during pregnancy (79, 91, 92).

##### Neuro-Cognitive Structure and Function

There is one case-control study investigated brain function in pre-term infants born to mother with GDM. In the first 33 days after delivery, the researchers used MRI image and discovered that infants born to mother with GDM tended to have multiple reduced fractional anisotropy in the brain, reflecting a microstructural white matter abnormalities compared with the infants born to mother without GDM (80).

#### GDM and Childhood Outcomes

Twenty studies on this topic were identified, with nearly half reported in China (n=8), then followed by India (n=4), Israel (n=3), Hong Kong (n=3), Pakistan (n=1), and Sri Lanka (n=1). Childhood outcomes spanned several traits and conditions, including adiposity and cadiometabolic outcomes, cognitive function, endocrinological and ophthalmological morbidity.

##### Anthropometry, Blood Pressure and Cardiometaboilc Outcomes

The majority of studies (17/20, 85.0%) reported consistent findings on long-term outcomes like childhood adiposity and cardio-metabolic risks. Overall, offspring born to women with GDM had higher BMI z-score, higher systolic blood pressure and diastolic blood pressure, higher childhood overweight and obesity rates, higher lipid profile levels, and higher insulin and insulin resistance levels, than those born to women without GDM. These studies involved small (n=164) to large (n=27 157) sample sizes of offspring with an average follow-up of 1-18 years among different ethnicities (Chinese, Indians, Sri Lankans and Israelite Jews).

In terms of cardiac function, we included one Pakistani study (93) and one Indian study (81) with small sample sizes of 136 and 236. Compared with their counterparts, offspring born to women with GDM had higher Carotid Intima-Media Thickness (cIMT), cardiac output and stroke volume, decreased mitral E/A ratio, and total peripheral resistance in early childhood and early adolescence, respectively.

Among Asian immigrants, two studies in the UK (94, 95) and one study in the US (96) with sample sizes ranging from 382 to 6 060 reported a consistent association between GDM and childhood obesity across all races and ethnic groups. The magnitude in such association between NHW women and Asian female immigrants was similar.

##### Neuro-Cognitive Outcomes

Hyperglycemia during pregnancy may affect fetal neurodevelopment and leave a significant impact on offspring cognition (97). Only one Indian study reported neurocognitive outcomes in the offspring at a mean 9.7 years of age (82). Children born to women with GDM had higher learning, long-term retrieval and storage, and better verbal ability than children born to women without GDM. The authors propose that the finding may be confounded by the strong correlation between GDM and higher social-economic status among this cohort (82).

##### Endocrinological and Ophthalmological Outcomes

Other childhood outcomes related to GDM include endocrine and ophthalmic morbidities. In two large-scale Israelite cohort studies where young adults (≤ 18 years) with a history of small-than-gestational age (SGA) conditions were recruited. One study showed no difference in the incidence of endocrine morbidity between young adults born to women with and without GDM (83). In contrast, the other study observed a higher prevalence of offspring ophthalmic inflammation (0.74% vs. 0.60%) and a 60% higher risk in ophthalmic-related hospitalization among young adults born to women with GDM and treated with medication (metformin, insulin) (78).

## Discussion and Future Direction

Our review reinforces that, in general, Asians are at the highest risk of developing GDM and for subsequent progression to T2D among all populations. Yet, data among the Asian population on long-term health implications of GDM on women and offspring remain limited and are less in-depth than the Western population. In addition, studies in identifying attributable risk factors that may inform preventive strategies of long-term adverse health outcomes among women and their offspring are less comprehensive in Asians than in the Western population. Methodologically, inferences from existing published data are hindered by considerable heterogeneity in study designs, a high risk of bias (**Supplementary Tables 1**, **2**), and standardized protocols for defining studies of Asians.

In order to address such critical knowledge gaps, future endeavors in the following aspects may be warranted to dissect the vicious circle of “diabetes begetting diabetes” and improve the health and well-being of this and future generations.

1. Conducting large scale well-designed cohort studies and/or consortium networks among Asians to investigate risk factors and etiology of GDM. A better understanding of GDM pathogenesis specific to Asian women shall further enhance our knowledge on the unique GDM characteristics among Asian women and develop more targeted and effective intervention approaches to prevent GDM and interrupt the transgenerational diabetic vicious cycle. However, such GDM heterogeneity-specific maternal health outcomes in Asians are still limited in scope, let alone other elements of the potential impact such as genetic factors and fetal sex. Future endeavors to establish parallel prospective pregnancy cohorts—with longitudinal data collection and comprehensive characterization of metabolic profiles through pregnancy in different Asian regions—are warranted to understand biological differences across Asian ethnicities, identify determinants and even develop prediction models for GDM onset and its phenotype-specific transgenerational health outcomes.

2. Conducting prospective cohort studies and/or intervention studies to follow up both GDM women and their offspring following the index pregnancy to identify factors that may mitigate the adverse impact of GDM on both women and their children. With the increasing awareness of the GDM burden and subsequent adverse health outcomes in Asian women and their offspring, a few large-scale ongoing pre-conception and pregnancy trials have focused on lifestyle intervention in Asia, such as Project SARAS in Mumbai (98) and the VINAVAC study in Vietnam (99). However, inferences from these two trials are inconsistent, which might be hindered by participants’ low compliance, including low uptake rate of OGTT, poor quality of data collection (e.g., physical examination, questionnaires administration) during research visits, and not quantitative constituents in the snack or freshly-prepared food given to the intervention group (98, 99). In terms of postpartum trials, substantial evidence in either lifestyle modifications (100) or pharmacological therapies (101–103) gathered from developed countries has shown promising results. However, intervention studies with customized approaches (e.g., diet recommendation, lifestyle modification) according to the Asian population are much fewer in scope than the Western population. Recently, there have been some improvements, including a few postpartum T2D prevention trials conducted in countries like China (100, 104), Singapore (105), Malaysia (106), and India (107), focusing on lifestyle modification, with a sample range between 77 and 1 414 and a length of follow-up up to 10 years. However, most of them are still ongoing, and only two trials reported more significant weight loss, reduction in waist circumference, and improved glucose tolerance during the 6-12 months’ postpartum period (104, 106).

3. Conducting studies of Health Disparities in GDM Care in Asian Populations across countries and continents. Even though developing countries in Asia (e.g., India) have shown increased life expectancy over the past several decades, health inequity is still a severe national issue as progress is uneven within each country (108). Furthermore, not all but a substantial proportion of Asian migrants in Western countries face socio-economical disadvantages such as access to health care and education (109). Among them, women seem to be more affected than men due to their vulnerability (109). Therefore, the fight against GDM and its harm to Asian mothers and children should account for existing health inequity and develop strategies to address health disparities.

4. Health Care System Improvement in Asia. Emerging evidence has pointed out that a portion of GDM cases was indeed overt diabetes that has not been identified before pregnancy, which ultimately drives the risk of maternal and offspring health outcomes even higher (110). For example, collecting information on pre-existing maternal diabetes or overt diabetes identification during early pregnancy in the Asian health care system is critical to screen for and even prevent offspring congenital abnormality or other adverse fetal and neonatal health outcomes. Ideally, GDM rates in the population could be reduced by individual and societal measures designed to promote healthy lifestyle changes, including optimal dietary intake and increased physical activity in the general population, focusing on the health and fitness of women of reproductive age.

## Data Availability Statement

The original contributions presented in the study are included in the article/**Supplementary Material**. Further inquiries can be directed to the corresponding authors.

## Author Contributions

L-JL contributed to the review’s framework conceptualization, study, design, literature research, data collection, analysis and interpretation, and manuscript write-up; LH contributed to literature search, data collection and summary; DT contributed to data interpretation and manuscript editing; CZ contributed to the review’s framework conceptualization, study design, data interpretation and manuscript editing. All authors contributed to the article and approved the submitted version.

## Funding

L-JL is funded by Singapore National Medical Research Council Clinician Science Award 2021 (NMRC CSAINV/002/2021).

## Conflict of Interest

The authors declare that the research was conducted in the absence of any commercial or financial relationships that could be construed as a potential conflict of interest.

## Publisher’s Note

All claims expressed in this article are solely those of the authors and do not necessarily represent those of their affiliated organizations, or those of the publisher, the editors and the reviewers. Any product that may be evaluated in this article, or claim that may be made by its manufacturer, is not guaranteed or endorsed by the publisher.

## Supplementary Material

Supplementary Figure 1 Flow diagram of search strategy and selection of GDM prevalence in the Asian population including Native Asians and Asian migrants.

Supplementary Figure 2 Flow diagram of search strategy and selection of GDM-related maternal postpartum health outcomes in the Asian population including Native Asians and Asian migrants.

Supplementary Figure 3 Flow diagram of search strategy and selection of GDM-related offspring postpartum health outcomes in the Asian population including Native Asians and Asian migrants.
