# Supplementary material for: Gestational Diabetes Mellitus Among Asians – A Systematic Review From a Population Health Perspective
Source: Front Endocrinol (Lausanne). 2022 Jun 16;13:840331. doi: 10.3389/fendo.2022.840331 (PMC9245567; doi:10.3389/fendo.2022.840331)
Supplement: Supplementary Figure 1 — Flow diagram of search strategy and selection of GDM prevalence in the Asian population including Native Asians and Asian migrants. [file DataSheet_1.docx]

Keywords searching: ((prevalence) OR (incidence) OR (gestational diabetes mellitus) OR (gestational diabetes) OR (diabetes in pregnancy)) AND ((Asia) OR (Asians) OR (Asian countries) OR (Asian population) OR (immigrants) OR (Asian migrants)) from Pubmed, Embase, Web of science and Scopus up till 30 June 2021 (n=5 772)

Screened by titles and removed duplicated studies, non-English article and studies on non-human subjects subject (n=2911)

Further screening by abstract (n=2861)

Excluded (n=2695)

1. Irrelevant (n=2618)
2. Review or meta-analysis (n=77)

Full-text articles assessed for eligibility (n=166)

Excluded (n=31)

1. Conference abstract (n=6)
2. Evaluating the value or applicability or feasibility of the criteria (n=9)
3. Reporting repeated or overlapping data (n=15)
4. Type 2 Diabetes (n=1)

Identified through manual searching via included studies/ references (n=40)

Studies finally included in this review (n=175), including:

Native Asians (n=147) & Asian migrants (n=28)

**Supplementary Figure 1. Flow diagram of search strategy and selection of GDM prevalence in the Asian population including Native Asians and Asian migrants**
