# Supplementary material for: Gestational Diabetes Mellitus Among Asians – A Systematic Review From a Population Health Perspective
Source: Front Endocrinol (Lausanne). 2022 Jun 16;13:840331. doi: 10.3389/fendo.2022.840331 (PMC9245567; doi:10.3389/fendo.2022.840331)
Supplement: Supplementary Figure 2 — Flow diagram of search strategy and selection of GDM-related maternal postpartum health outcomes in the Asian population including Native Asians and Asian migrants. [file DataSheet_2.docx]

Keywords searching: ((gestational diabetes mellitus) OR (gestational diabetes) OR (diabetes in pregnancy)) AND ((Type 2 diabetes) OR (prediabetes) OR (glucose intolerance) OR (abnormal glucose) OR (hypertension) OR (high blood pressue) OR (cardiovascular disease) OR (kidney disease) OR (cancer) OR (liver dysfunction) OR (non-alcoholic fatty acid disease) OR (NAFLD) OR (health outcomes)) AND ((after delivery) OR (postpartum)) from Pubmed, Embase, Web of science and Scopus up till 30 June 2021 (n=1 107)

Screened by titles and removed duplicated studies, non-English article and studies on non-human subjects subject (n=440)

Further screening by abstract (n=667)

Excluded (n=580)

1. Irrelevant (n=560)
2. Review or meta-analysis or Book (n=19)
3. Case report (n=1)

Full-text articles assessed for eligibility (n=87)

Excluded (n=21)

1. Conference abstract (n=18)
2. Reporting repeated or overlapping data (n=3)

Identified through manual searching via included studies/ references (n=8)

Studies finally included in this review (n=74), including:

Native Asians (n=72) & Asian migrants (n=2)

**Supplementary Figure 2. Flow diagram of search strategy and selection of GDM-related maternal postpartum health outcomes in the Asian population including Native Asians and Asian migrants**
