# Supplementary material for: Gestational Diabetes Mellitus Among Asians – A Systematic Review From a Population Health Perspective
Source: Front Endocrinol (Lausanne). 2022 Jun 16;13:840331. doi: 10.3389/fendo.2022.840331 (PMC9245567; doi:10.3389/fendo.2022.840331)
Supplement: Supplementary Figure 3 — Flow diagram of search strategy and selection of GDM-related offspring postpartum health outcomes in the Asian population including Native Asians and Asian migrants. [file DataSheet_3.docx]

Keywords searching: ((gestational diabetes mellitus) OR (gestational diabetes) OR (diabetes in pregnancy)) AND ((cardio-metabolic outcome) OR (cognitive outcome) OR (congenital disease) OR (adiposity) OR (hypertension) OR (health outcome) OR (neuro-cognitive outcome) OR (obesity) OR (diabetes) OR (cardiovascular disease) OR (kidney disease) OR (cancer)) AND ((child) OR (offspring)) from Pubmed, Embase, Web of science and Scopus up till 30 June 2021 (n=596)

Screened by titles and removed duplicated studies, non-English article and studies on non-human subjects subject (n=295)

Further screening by abstract (n=301)

Excluded (n=280)

1. Irrelevant (n=264)
2. Review or meta-analysis or Book or Protocol (n=16)

Full-text articles assessed for eligibility (n=21)

Excluded (n=6)

1. Conference abstract (n=3)
2. Reporting repeated or overlapping data (n=3)

Identified through manual searching via included studies/ references (n=35)

Studies finally included in this review (n=50), including:

Native Asians (n=42) & Asian migrants (n=8)

**Supplementary Figure 3. Flow diagram of search strategy and selection of GDM-related offspring postpartum health outcomes in the Asian population including Native Asians and Asian migrants**
