# Supplementary material for: Gestational Diabetes Mellitus Among Asians – A Systematic Review From a Population Health Perspective
Source: Front Endocrinol (Lausanne). 2022 Jun 16;13:840331. doi: 10.3389/fendo.2022.840331 (PMC9245567; doi:10.3389/fendo.2022.840331)
Supplement: Supplementary file 4 [file DataSheet_4.docx]

**Supplementary Table 1. NOSC for GDM and maternal health outcomes in Asian Native and Asian migrants studies**

| **Study** | | **Selection (4)** | | | | **Comparability (2)** | | **Outcomes (3)** | | | **Total (9)** | **Risk of bias** |
| --- | --- | --- | --- | --- | --- | --- | --- | --- | --- | --- | --- | --- |
|  |  | Representative of exposed cohort | Selection of non-exposed cohort | Ascertainment of exposure | Outcome not present at start of the study | Main factor | Additional factor | Assessment of outcomes | Clearly described and appropriate statistical test | Adequate follow-up length + follow-up rate |  |  |
| Native Asian studies | | | | | | | | | | | | |
| 1 | Pei et al. | * |  | * |  | * | * | * | * |  | 6 | High |
| 2 | Mao et al. | * |  |  |  | * |  | * | * |  | 4 | High |
| 3 | Miao et al. |  |  | * | * | * |  | * | * | * | 6 | High |
| 4 | Wang et al. | * |  | * | * | * |  | * | * |  | 6 | High |
| 5 | Liu et al. |  |  | * | * | * |  | * | * |  | 5 | High |
| 6 | Fan et al. | * |  | * | * | * |  | * | * | * | 7 | Low |
| 7 | Ma et al. | * |  | * | * | * |  | * |  |  | 6 | High |
| 8 | Mai et al. |  |  | * |  | * | * | * |  | * | 5 | High |
| 9 | Chang et al. |  |  | * | * | * |  |  |  |  | 3 | Very high |
| 10 | Cao et al. |  |  | * | * | * |  | * | * |  | 5 | High |
| 11 | Lin et al. |  |  | * |  | * |  | * |  | * | 4 | High |
| 12 | Shek et al. |  | * | * | * | * |  | * |  | * | 6 | High |
| 13 | Tam et al. |  |  | * | * | * | * | * |  | * | 6 | High |
| 14 | Lee et al. | * |  | * | * | * |  | * |  | * | 6 | High |
| 15 | Ko et al. | * |  | * | * | * |  | * |  |  | 5 | High |
| 16 | Kawasaki et al. | * |  | * |  | * |  | * | * | * | 6 | High |
| 17 | Kasuga et al. | * |  | * | * | * |  | * |  |  | 5 | High |
| 18 | Inoue et al. |  |  | * | * | * |  | * |  | * | 5 | High |
| 19 | Kondo et al. |  |  | * |  | * |  | * |  |  | 3 | Very high |
| 20 | Kugishima et al. | * |  | * |  | * |  | * |  | * | 5 | High |
| 21 | Nishikawa et al. |  |  | * | * | * |  | * |  |  | 4 | High |
| 22 | Yasuhi et al. |  |  | * |  | * |  | * |  | * | 4 | High |
| 23 | Kugishima et al. | * |  | * |  | * |  | * |  |  | 4 | High |
| 24 | Han et al. | * | * | * |  | * | * | * | * | * | 8 | Low |
| 25 | Cho et al. | * |  | * | * | * | * | * | * | * | 8 | Low |
| 26 | Cho et al. | * | * | * | * | * |  | * | * | * | 8 | Low |
| 27 | Kim et al. | * | * | * | * | * |  | * |  |  | 6 | High |
| **Study** | | **Selection (4)** | | | | **Comparability (2)** | | **Outcomes (3)** | | | **Total (9)** | **Risk of bias** |
|  |  | Representative of exposed cohort | Selection of non-exposed cohort | Ascertainment of exposure | Outcome not present at start of the study | Main factor | Additional factor | Assessment of outcomes | Clearly described and appropriate statistical test | Adequate follow-up length + follow-up rate |  |  |
| 28 | Shin et al. | * |  | * | * | * |  | * | * |  | 6 | High |
| 29 | Cho et al. | * | * | * |  | * |  | * |  |  | 5 | High |
| 30 | Moon et al. |  |  | * | * | * |  | * |  | * | 5 | High |
| 31 | Yang et al. |  |  | * | * | * |  | * |  | * | 5 | High |
| 32 | Kwak et al. | * |  | * | * | * |  | * |  | * | 6 | High |
| 33 | Kwak et al. | * |  | * | * | * |  | * |  | * | 6 | High |
| 34 | Kim et al. | * |  | * | * | * |  | * |  | * | 6 | High |
| 35 | Lee et al. | * | * | * | * | * |  | * |  | * | 7 | Low |
| 36 | Lim et al. |  |  | * | * | * |  | * |  |  | 4 | High |
| 37 | Cho et al. | * | * | * | * | * |  | * |  | * | 7 | Low |
| 38 | Jang et al. | * |  | * | * | * |  | * |  |  | 5 | High |
| 39 | Ruksasakul et al. |  | * | * |  | * | * | * |  | * | 6 | High |
| 40 | Youngwanichsetha et al. |  |  | * | * | * |  | * |  |  | 4 | High |
| 41 | Chew et al. |  | * | * |  | * |  | * |  | * | 5 | High |
| 42 | Hewage et al. |  |  | * | * | * |  | * |  | * | 5 | High |
| 43 | Malong et al. |  |  | * | * | * |  | * |  | * | 5 | High |
| 44 | Goyal et al. | * |  | * | * | * |  | * |  | * | 6 | High |
| 45 | Bhavadharini et al. |  |  | * | * | * |  | * |  |  | 4 | High |
| 46 | Gupta et al. | * |  | * | * | * |  | * |  | * | 6 | High |
| 47 | Jindal et al. |  |  | * | * | * |  | * |  |  | 4 | High |
| 48 | Mahalakshmi et al. |  |  | * |  | * |  | * |  | * | 4 | High |
| 49 | Krishnaveni et al., |  |  | * |  | * |  | * |  | * | 5 | High |
| 50 | Sudasinghe et al. |  |  | * | * | * |  | * |  | * | 5 | High |
| 51 | Herath et al. |  |  | * | * | * |  | * |  | * | 5 | High |
| 52 | Wijeyaratne et al. |  |  | * | * | * |  | * |  | * | 5 | High |
| 53 | Aziz et al. |  |  | * | * | * |  | * |  | * | 5 | High |
| 54 | Yefet et al. | * |  | * | * | * |  | * |  | * | 6 | High |
| 55 | Chodick et al. | * | * | * |  | * |  | * | * | * | 7 | Low |
| **Study** | | **Selection (4)** | | | | **Comparability (2)** | | **Outcomes (3)** | | | **Total (9)** | **Risk of bias** |
|  |  | Representative of exposed cohort | Selection of non-exposed cohort | Ascertainment of exposure | Outcome not present at start of the study | Main factor | Additional factor | Assessment of outcomes | Clearly described and appropriate statistical test | Adequate follow-up length + follow-up rate |  |  |
| 56 | Kerimoglu et al. |  |  | * |  | * |  | * |  |  | 3 | Very high |
| 57 | Minooee et al. | * |  | * | * | * |  | * |  | * | 6 | High |
| 58 | Nouhjah et al. |  |  | * | * | * |  | * |  |  | 4 | High |
| 59 | Valizadeh et al. |  |  | * | * | * | * | * |  | * | 6 | High |
| 60 | Hossein-Nezhad et al. |  |  | * |  | * |  | * |  |  | 3 | Very high |
| 61 | Agarwal et al. | * |  | * |  | * |  | * |  |  | 4 | High |
| 62 | Wahabi et al. |  |  | * | * | * |  | * |  | * | 5 | High |
| 63 | Mahzari et al. |  |  | * |  | * |  | * |  |  | 3 | Very high |
| 64 | Peng et al. | * | * | * |  | * |  | * | * | * | 7 | Low |
| 65 | Fuchs et al. | * | * | * |  | * |  | * | * | * | 7 | Low |
| 66 | Sella et al. | * | * | * |  | * |  | * | * | * | 7 | Low |
| 67 | Perrin et al | * |  | * |  | * |  | * | * | * | 6 | High |
| 68 | Perrin et al. | * |  | * |  | * |  | * | * | * | 6 | High |
| 69 | Wang et al. | * |  | * | * | * |  | * | * | * | 7 | Low |
| 70 | Shen et al. |  |  |  |  |  |  |  |  |  |  |  |
| 71 | Kessous et al. | * | * | * | * | * |  | * |  | * | 7 | Low |
| 72 | Kubihal et al. |  |  | * |  | * |  | * |  | * | 4 | High |
| Asian migrant studies | | | | | | | | | | | | |
| 1 | Prados et al |  |  | * | * | * |  | * |  | * | 5 | High |
| 2 | Mekerji et al. | * | * | * | * | * |  | * |  | * | 7 | Low |
