# Supplementary material for: Gestational Diabetes Mellitus Among Asians – A Systematic Review From a Population Health Perspective
Source: Front Endocrinol (Lausanne). 2022 Jun 16;13:840331. doi: 10.3389/fendo.2022.840331 (PMC9245567; doi:10.3389/fendo.2022.840331)
Supplement: Supplementary file 5 [file DataSheet_5.docx]

**Supplementary Table 2. NOSC for GDM and offspring health outcomes in Asian Native and Asian migrants studies**

| **Study** | | **Selection (4)** | | | | **Comparability (2)** | | **Outcomes (3)** | | | **Total (9)** | **Risk of bias** |
| --- | --- | --- | --- | --- | --- | --- | --- | --- | --- | --- | --- | --- |
|  |  | Representative of exposed cohort | Selection of non-exposed cohort | Ascertainment of exposure | Outcome not present at start of the study | Main factor | Additional factor | Assessment of outcomes | Clearly described and appropriate statistical test | Adequate follow-up length + follow-up rate |  |  |
| Native Asian studies8 | | | | | | | | | | | | |
| 1 | Venkataraman et al. |  | * | * | * | * |  | * | * | * | 7 | Low |
| 2 | Hu et al. | * | * | * | * | * |  | * | * | * | 8 | Low |
| 3 | Yan et al. | * | * | * | * | * |  | * | * | * | 8 | Low |
| 4 | Cheng et al. |  | * | * | * | * |  | * | * | * | 9 | Low |
| 5 | Yang et al. | * | * | * | * | * |  | * | * | * | 8 | Low |
| 6 | Ding et al. |  |  | * |  | * |  | * |  | * | 4 | Very high |
| 7 | Wang et al. | * | * | * |  | * |  | * | * | * | 7 | Low |
| 8 | Zhao et al. |  | * | * | * | * |  | * |  | * | 6 | High |
| 9 | Wang et al. |  | * | * | * | * |  | * |  | * | 6 | High |
| 10 | Chen et al. |  |  | * | * |  |  | * | * | * | 5 | High |
| 11 | Mannan et al. |  | * | * |  | * |  | * |  | * | 5 | High |
| 12 | Jang et al. |  | * | * |  | * |  | * |  | * | 5 | High |
| 13 | Groof et al. |  | * | * |  | * |  | * | * | * | 6 | High |
| 14 | Riskin et al., |  | * | * |  | * |  | * |  | * | 5 | High |
| 15 | Walter et al. | * | * | * |  | * |  | * |  | * | 6 | High |
| 16 | Samsudddin et al. |  | * | * | * | * |  | * |  | * | 6 | High |
| 17 | Alfadhli et al. |  | * | * | * | * |  | * | * | * | 7 | Low |
| 18 | Luengmettakul et al. |  | * | * |  | * | * | * | * | * | 7 | Low |
| 19 | Youngwanichesetha et al. |  |  | * | * | * |  | * |  | * | 5 | High |
| 20 | Mahalakshmi et al. |  |  | * |  | * |  | * |  | * | 4 | High |
| 21 | Vijay et al. |  | * | * |  | * |  | * |  | * | 5 | High |
| 22 | Soylu et al. |  | * | * |  | * |  | * |  | * | 5 | High |
| 23 | Tain et al. | * | * | * |  | * |  | * | * | * | 7 | Low |
| 24 | Liu et al. |  | * | * |  | * |  | * | * | * | 6 | High |
| 25 | Xuan et al. |  | * | * |  | * |  | * |  | * | 5 | High |
| 26 | Du et al., |  | * | * | * | * |  | * | * |  | 6 | High |
| 27 | Liang et al. | * | * | * |  | * |  | * |  | * | 6 | High |
| **Study** | | **Selection (4)** | | | | **Comparability (2)** | | **Outcomes (3)** | | | **Total (9)** | **Risk of bias** |
|  |  | Representative of exposed cohort | Selection of non-exposed cohort | Ascertainment of exposure | Outcome not present at start of the study | Main factor | Additional factor | Assessment of outcomes | Clearly described and appropriate statistical test | Adequate follow-up length + follow-up rate |  |  |
| 28 | Wang et al. | * | * | * | * | * |  | * |  | * | 7 | Low |
| 29 | Zhang et al. |  | * | * |  | * |  | * |  | * | 5 | High |
| 30 | Chang et al. | * | * | * |  | * |  | * |  | * | 6 | High |
| 31 | Liu et al. | * | * | * | * | * |  | * |  | * | 7 | Low |
| 32 | Andegiorgish et al. |  | * | * |  | * |  | * |  | * | 5 | High |
| 33 | Hui et al. | * | * | * | * | * |  | * | * | * | 8 | Low |
| 34 | Tam et al. |  | * | * | * | * |  | * |  | * | 6 | High |
| 35 | Tam et al. |  | * | * | * | * |  | * |  | * | 6 | High |
| 36 | Krishnaveni et al. |  | * | * | * | * |  | * |  | * | 6 | High |
| 37 | Krishnaveni et al. |  | * | * | * | * |  | * |  | * | 6 | High |
| 38 | Tsadok et al. |  | * | * | * | * |  | * |  | * | 6 | High |
| 39 | Herath et al. |  | * | * |  | * |  | * | * | * | 6 | High |
| 40 | Hoodbhoy et al. |  | * | * |  | * |  | * | * | * | 6 | High |
| 41 | Veena et al. |  | * | * |  | * |  | * |  | * | 5 | High |
| 42 | Shorer et al. | * | * | * |  | * |  | * | * | * | 7 | Low |
| Asian migrant studies | | | | | | | | | | | | |
| 1 | Slenter et al. |  | * | * | * | * |  | * | * | * | 7 | Low |
| 2 | Anand et al. |  | * | * | * | * |  | * |  | * | 6 | High |
| 3 | Kosman et al. |  | * | * | * | * |  | * |  | * | 6 | High |
| 4 | Bower et al. | * | * | * |  | * |  | * |  | * | 6 | High |
| 5 | Mocarski et al. | * | * | * |  | * |  | * |  | * | 6 | High |
| 6 | Faith et al. |  |  | * | * | * |  | * |  | * | 5 | High |
| 7 | West et al. |  | * | * | * | * |  | * |  | * | 5 | High |
| 8 | Fairley et al. |  | * | * | * | * |  | * |  | * | 5 | High |
