# Supplementary material for: Gestational Diabetes Mellitus Among Asians – A Systematic Review From a Population Health Perspective
Source: Front Endocrinol (Lausanne). 2022 Jun 16;13:840331. doi: 10.3389/fendo.2022.840331 (PMC9245567; doi:10.3389/fendo.2022.840331)
Supplement: Supplementary file 6 [file DataSheet_6.docx]

**Supplementary Table 3. Summary of GDM prevalence in native Asian women study**

| **Region of Asia** | **Country** | **No** | **PMID/DOI/weblink** | **Author** | **Year** | **GDM diagnostic method** | **Study setting** | **One- or Two-Step** | **GCT-details** | **Sample size** | **GDM cases** | **GDM Prevalence (%)** |
| --- | --- | --- | --- | --- | --- | --- | --- | --- | --- | --- | --- | --- |
| East Asia | China | 1 | 31525546 | Zhang et al., | 2020 | IADPSG | Hospital-based | One-step | N.A. | 5,165 | 604 | 11.7 |
|  |  | 2 | 32316796 | Wang et al., | 2020 | IADPSG | Hospital-based | one-step | N.A. | 26,301 | 6,376 | 24.2 |
|  |  | 3 | 31192561 | Huang et al., | 2019 | IADPSG | Hospital-based | One-step | N.A. | 326 | 33 | 10.1 |
|  |  | 4 | 30572275 | Zhou et al., | 2019 | IADPSG | Hospital-based | One-step | N.A. | 3,300 | 378 | 11.5 |
|  |  | 5 | 30769927 | Hu et al., | 2019 | IADPSG | Hospital-based | One-step | N.A. | 1,014 | 238 | 23.5 |
|  |  | 6 | 31731641 | Cheng et al., | 2019 | IADPSG | Hospital-based | One-step | N.A. | 950 | 97 | 10.2 |
|  |  | 7 | 31626126 | Wang et al., | 2019 | IADPSG | Hospital-based | One-step | N.A. | 2,698 | 462 | 17.1 |
|  |  | 8 | 30732876 | Zhu et al., | 2019 | IADPSG | Hospital-based | One-step | N.A. | 3,110 | 399 | 12.8 |
|  |  | 9 | 30844582 | Liu et al., | 2019 | IADPSG | Hospital-based | One-step | N.A. | 1,087 | 103 | 9.5 |
|  |  | 10 | 30869198 | Yan et al., | 2019 | IADPSG | Community-based | One-step | N.A. | 78,572 | 13,846 | 17.6 |
|  |  | 11 | 31271809 | Yang et al., | 2019 | IADPSG | Community-based | Two-step | 50g 1-hour glucose level >= 7.8 mmol/L | 19,622 | 1,495 | 7.6 |
|  |  | 12 | 30454043 | Mak et al., | 2018 | IADPSG | Hospital-based | One-step | N.A. | 1,337 | 199 | 14.9 |
|  |  | 13 | 30159334 | Li et al., | 2018 | IADPSG | Hospital-based | One-step | N.A. | 6,941 | 1,020 | 14.7 |
|  |  | 14 | 29425094 | Liu et al., | 2018 | IADPSG | Hospital-based | One-step | N.A. | 2,026 | 198 | 9.8 |
|  |  | 15 | 29384326 | Wu et al., | 2018 | IADPSG | Hospital-based | One-step | N.A. | 4,959 | 1,080 | 21.8 |
|  |  | 16 | 30030928 | Schaefer et al., | 2018 | IADPSG | Hospital-based | One-step | N.A. | 8,381 | 1,129 | 13.5 |
|  |  | 17 | 29907325 | Yang et al., | 2018 | IADPSG | Hospital-based | One-step | N.A. | 1,232 | 234 | 19.0 |
|  |  | 18 | 29292753 | Xu et al., | 2018 | IADPSG | Hospital-based | One-step | N.A. | 2,345 | 87 | 3.7 |
|  |  | 19 | 28636764 | Song et al., | 2017 | IADPSG | Hospital-based | One-step | N.A. | 6,886 | 1,005 | 14.6 |
|  |  | 20 | 29335058 | Du et al., | 2017 | IADPSG | Hospital-based | One-step | N.A. | 753 | 64 | 8.5 |
|  |  | 21 | 28469095 | Zhu et al., | 2017 | China MOH | Hospital-based | One-step | N.A. | 15,194 | 2,987 | 19.7 |
|  |  | 22 | 27079350 | Li et al., | 2016 | IADPSG | Hospital-based | One-step | N.A. | 327 | 48 | 14.7 |
|  |  | 23 | 27264612 | Leng et al., | 2016 | IADPSG | Community-based | Two-step | 50g 1-hour glucose level >= 7.8 mmol/L | 17359 | 1,332 | 7.7 |
|  |  | 24 | 25820620 | Zhang et al., | 2015 | IADPSG | Community-based | Two-step | 50g 1-hour glucose level >= 7.8 mmol/L | 14,198 | 1,069 | 7.5 |
|  |  | 25 | 24232664 | Shang et al., | 2014 | IADPSG | Hospital-based | One-step | N.A. | 3,083 | 612 | 19.9 |
|  |  | 26 | 25271112 | Chang et al., | 2014 | ADA 2007 | Hospital-based | one-step | N.A. | 28,434 | 2,400 | 8.4 |
|  |  | 27 | 23920137 | Li et al., | 2014 | ADA 2012 | Hospital-based | One-step | N.A. | 539 | 69 | 12.8 |
|  |  | 28 | 21569085 | Zhang et al., | 2011 | WHO 1999 | Hospital-based | Two-step | 50g 1-hour glucose level >= 7.8 mmol/L | 105,473 | 4,764 | 4.5 |
|  |  | 29 | 19929987 | Yang et al., | 2009 | ADA 2004 | Hospital-based | Two-step | 50g 1-hour glucose level >= 7.8 mmol/L | 16,286 | 708 | 4.3 |
|  |  | 30 | 11978679 | Yang et al., | 2002 | WHO 1998 | Hospital-based | Two-step | 50g 1-hour glucose level >= 7.8 mmol/L | 9286 | 177 | 1.9 |
|  | Hong Kong | 31 | 26492926 | Cheuk et al., | 2016 | WHO 1999 | Hospital-based | One-step | N.A. | 520 | 169 | 32.5 |
|  |  | 32 | 11869309 | Ko et al., | 2002 | WHO 1998 | Hospital-based | one-step | N.A. | 942 | 134 | 14.2 |
|  | Taiwan | 33 | 31982755 | Su et al., | 2020 | IADPSG | Community-based | One-step | N.A. | 371,131 | 43,538 | 11.7 |
|  |  |  |  |  |  | CC |  |  |  |  |  |  |
|  |  | 34 | 25614928 | Lin et al., | 2015 | ADA 2003 | Hospital-based | One-step | N.A. | 132 | 51 | 38.6 |
|  | South Korea | 35 | 33316311 | Jung et al., | 2020 | ICD | Community-based | Two-step | 50g 1-hour glucose level >= 7.8 mmol/L | 61 654 | 7956 | 12.9 |
|  |  | 36 | 27764258 | Koo et al., | 2016 | ICD | Community-based | Two-step | 50g 1-hour glucose level >= 7.8 mmol/L | 1,306,281 | 98,403 | 7.5 |
|  |  | 37 | 26292282 | Cho et al., | 2015 | ICD-10 | Community-based | Two-step | 50g | 1,824,913 | 129,666 | 7.1 |
|  |  | 38 | 23682224 | Yang et al., | 2013 | CC | Hospital-based | Two-step | 50g 1-hour glucose level >= 7.8 mmol/L | 1,163 | 269 | 23.1 |
|  |  | 39 | 9686918 | Jang et al, | 1998 | NDDG | Hospital-based | Two-step | 50g 1-hour glucose level >= 7.8 mmol/L | 9,005 | 173 | 1.9 |
|  | Japan | 40 | 32506178 | Dong et al., | 2020 | Japan Diabetes Society | Community-based | Two-step | 50g 1-hour glucose level >= 7.8 mmol/L | 84,948 | 1,904 | 2.2 |
|  |  | 41 | 30897272 | Iwama et al., | 2019 | IADPSG | Hospital-based | One-step | N.A. | 2,578 | 149 | 5.8 |
|  |  | 42 | 27025793 | Shimodaira et al., | 2016 | ADA 2004 | Hospital-based | Two-step | 50g 1-hour glucose level >= 7.8 mmol/L | 5,424 | 149 | 2.7 |
|  |  | 43 | 23292170 | Saisho et al., | 2013 | Japan Diabetes Society | Hospital-based | Two-step | 50g 1-hour glucose level >= 7.8 mmol/L | 62 | 15 | 24.2 |
|  |  | 44 | 14581157 | Maegawa et al., | 2003 | Japan Diabetes Society | Hospital-based | Two-step | 50g 1-hour glucose level >= 7.8 mmol/L | 749 | 22 | 2.9 |
| South East Asia | Singapore | 45 | 27657116 | De Seymour et al., | 2016 | WHO 1999 | Hospital-based | One-step | N.A. | 909 | 160 | 17.6 |
|  |  | 46 | doi:10.1111/14710528.13384 | Li et al., | 2015 | WHO 1999 | Hospital-based | One-step | N.A. | 5,739 | 578 | 10.1 |
|  |  | 47 | 24936548 | Yew et al., | 2014 | WHO 2013 | Hospital-based | One-step | N.A. | 855 | 180 | 21.1 |
|  |  |  |  |  |  | WHO 1999 | Hospital-based | One-step | N.A. | 855 | 246 | 28.8 |
|  |  | 48 | 25273851 | Chong et al., | 2014 | WHO 1999 | Hospital-based | One-step | N.A. | 1136 | 215 | 18.9 |
|  | Thailand | 49 | https://he02.tci-thaijo.org/index.php/tjog/article/view/22904/32868 | Suntorn et al., | 2015 | IADPSG | Hospital-based | One-step | N.A. | 325 | 71 | 21.8 |
|  |  | 50 | 26372349 | Srichumchit et al., | 2015 | NDDG | Hospital-based | Two-step | 50g 1-hour glucose level >= 7.8 mmol/L | 21,771 | 1,350 | 6.2 |
|  |  | 51 | http://citeseerx.ist.psu.edu/viewdoc/download?doi=10.1.1.931.7235&rep=rep1&type=pdf | Warunpitkul et al., | 2014 | CC | Hospital-based | Two-step | 50g 1-hour glucose level >= 7.8 mmol/L | 1,363 | 340 | 24.9 |
|  |  | 52 | 8877291 | Deerochanawong et al., | 1996 | WHO 1985 | Hospital-based | Two-step | 50g 1-hour glucose level >= 7.8 mmol/L | 709 | 111 | 15.7 |
|  | Vietnam | 53 | 29948409 | Nguyen et al., | 2018 | WHO 2013 | Hospital-based | One-step | N.A. | 1,987 | 432 | 22.8 |
|  |  | 54 | 22911157 | Hirst et al., | 2012 | IADPSG | Hospital-based | One-step | N.A. | 2,702 | 550 | 20.4 |
|  | Malaysia | 55 | 31923230 | Yong et al., | 2020 | Malaysia MOH guideline | Hospital-based | One-step | N.A. | 452 | 48 | 10.6 |
|  |  | 56 | 19154997 | Tan et al., | 2009 | WHO 1999 | Hospital-based | Two-step | 50g 1-hour glucose level >= 7.2mmol/L | 1,368 | 168 | 12.3 |
|  |  | 57 | 11597616 | Shamsuddin et al., | 2001 | WHO 1985 | Hospital-based | One-step | N.A. | 768 | 191 | 24.9 |
| South Asia | India | 58 | 30508848 | Basu et al., | 2020 | DIPSI | Hospital-based | One-step | N.A. | 735 | 127 | 17.2 |
|  |  | 59 | doi:10.1007/s13410-020-00798-4 (2020) | Panigrahi et al., | 2020 | DIPSI | Hospital-based | One-step | N.A. | 218 | 30 | 13.8 |
|  |  | 60 | doi:10.1007/s13410-018-0635-0 (2019). | Basha et al., | 2019 | WHO 2013 | Hospital-based | One-step | N.A. | 644 | 87 | 13.5 |
|  |  | 61 | 31311218 | Rajasekar et al., | 2019 | IADPSG | Hospital-based | One-step | N.A. | 630 | 88 | 14.0 |
|  |  | 62 | 29545111 | Agarwal et al., | 2018 | IADPSG | Hospital-based | One-step | N.A. | 6,520 | 1,193 | 18.3 |
|  |  | 63 | doi:10.7860/JCDR/2018/36575.11915 (2018) | Agarwal et al., | 2018 | IADPSG | Hospital-based | One-step | N.A. | 5,855 | 814 | 13.9 |
|  |  | 64 | https://www.pravara.com/pmr/pmr-9-3-2.pdf | Satyajit et al., | 2017 | DIPSI | Community-based | One-step | N.A. | 500 | 26 | 5.2 |
|  |  | 65 | [http://dx.doi.org/10.18203/2320-1770.ijrcog20172926](https://dx.doi.org/10.18203/2320-1770.ijrcog20172926) | Jadhav et al., | 2017 | DIPSI | Hospital-based | One-step | N.A. | 1,000 | 80 | 8.0 |
|  |  | 66 | DOI:[10.18203/2349-3933.ijam20172605](http://dx.doi.org/10.18203/2349-3933.ijam20172605) | Makwana et al., | 2017 | DIPSI | Hospital-based | One-step | N.A. | 476 | 38 | 8.0 |
|  |  | 67 | doi:10.14260/jemds/2017/111 | Vareed et al., | 2017 | WHO 2013 | Hospital-based | One-step | N.A. | 135 | 16 | 11.9 |
|  |  | 68 | [http://dx.doi.org/10.18203/2320-1770.ijrcog20160081](https://dx.doi.org/10.18203/2320-1770.ijrcog20160081) | Gracelyn et al., | 2016 | ADA 2014 | Hospital-based | One-step | N.A. | 500 | 59 | 11.8 |
|  |  | 69 | 26991305 | Nielsen et al., | 2016 | DIPSI | Hospital-based | One-step | N.A. | 4,053 | 659 | 16.3 |
|  |  | 70 | 28702243 | Bhavadharini et al., | 2016 | IADPSG | Hospital-based | One-step | N.A. | 1,774 | 278 | 15.7 |
|  |  | 71 | [http://dx.doi.org/10.18203/2320-1770.ijrcog20161707](https://dx.doi.org/10.18203/2320-1770.ijrcog20161707) | Sharma et al., | 2016 | IADPSG | Hospital-based | One-step | N.A. | 417 | 74 | 17.7 |
|  |  | 72 | https://www.ijrcog.org/index.php/ijrcog/article/view/1069/985 | Thathagari et al., | 2016 | NDDG | Hospital-based | Two-step | 50g 1-hour glucose level >= 7.8 mmol/L | 800 | 42 | 5.3 |
|  |  |  |  |  |  |  |  |  |  |  |  |  |
|  |  | 73 | 26476488 | Mohan et al., | 2016 | WHO 2013 | Hospital-based | Two-step | 50g 1-hour glucose level >= 7.8 mmol/L | 201 | 32 | 15.9 |
|  |  | 74 | 26012589 | Arora et al., | 2015 | WHO 1999 | Hospital-based | One-step | N.A. | 5,100 | 458 | 9.0 |
|  |  |  |  |  |  | WHO 2013 | Hospital-based | One-step | N.A. |  | 1,779 | 35.0 |
|  |  | 75 | DOI:[10.18203/2320-1770.ijrcog20151271](http://dx.doi.org/10.18203/2320-1770.ijrcog20151271) | Shridevi et al., | 2015 | DIPSI | Hospital-based | Two-step | 50g 1-hour glucose level >= 7.8 mmol/L | 200 | 23 | 11.5 |
|  |  | 76 | 26180767 | Bhatt et al., | 2015 | DIPSI | Community-based | One-step | N.A. | 989 | 94 | 9.5 |
|  |  | 77 | https://www.bibliomed.org/mnsfulltext/89/89-1442996892.pdf?1595946755 | Swaroop et al., | 2015 | DIPSI | Hospital-based | One-step | N.A. | 225 | 22 | 9.8 |
|  |  | 78 | 26119433 | Gopalakrishnan et al., | 2015 | IADPSG | Hospital-based | One-step | N.A. | 332 | 139 | 41.9 |
|  |  | 79 | doi:10.1016/j.cegh.2013.10.001 | Goswami et al., | 2014 | WHO 1985 | Hospital-based | One-step | N.A. | 930 | 28 | 3.0 |
|  |  | 80 | https://www.ijrcog.org/index.php/ijrcog/article/view/823/768 | Kalyani et al., | 2014 | WHO 1999 | Hospital-based | One-step | N.A. | 300 | 25 | 8.3 |
|  |  | 81 | 24944930 | Rajput et al., | 2014 | WHO 1999 | Community-based | One-step | N.A. | 913 | 127 | 13.9 |
|  |  | 82 | DOI:[10.5455/ijmsph.2013.211120131](http://dx.doi.org/10.5455/ijmsph.2013.211120131) | Raja et al., | 2013 | DIPSI | Community-based | One-step | N.A. | 306 | 24 | 7.8 |
|  |  | 83 | 23961485 | Kalra et al., | 2013 | DIPSI | Hospital-based | One-step | N.A. | 500 | 33 | 6.6 |
|  |  | 84 | 24290085 | Nayak et al., | 2013 | IADPSG | Hospital-based | One-step | N.A. | 304 | 83 | 27.3 |
|  |  | 85 | 23703340 | Rajput et al., | 2013 | ADA 2004 | Hospital-based | One-step | N.A. | 607 | 43 | 7.1 |
|  |  | 86 | DOI:[10.14260/jemds/490](http://dx.doi.org/10.14260/jemds/490) | Singh and Uma | 2013 | DIPSI | Hospital-based | One-step | N.A. | 400 | 23 | 5.7 |
|  |  | 87 | 22195364 | Jali et al., | 2011 | WHO 1998 | Hospital-based | One-step | N.A. | 325 | 52 | 16.0 |
|  |  | 88 | 21755759 | Wahi et al., | 2011 | DIPSI | Hospital-based | One-step | N.A. | 2,025 | 132 | 6.5 |
|  |  | 89 | 22176476 | Tripathi et al., | 2011 | CC | Hospital-based | Two-step | 50g 1-hour glucose level >= 7.8 mmol/L | 700 | 10 | 1.4 |
|  |  | 90 | 19154999 | Seshiah et al., | 2009 | WHO 1994 | Community-based | One-step | N.A. | 12,056 | 1,679 | 13.9 |
|  |  | 91 | 19368095 | Swami et al., | 2008 | ADA 2005 | Hospital-based | Two-step | 50g 1-hour glucose level >= 7.8 mmol/L | 1,225 | 94 | 7.7 |
|  |  | 92 | 17640759 | Krishnaveni et al., | 2007 | CC | Hospital-based | One-step | N.A. | 524 | 21 | 4.0 |
|  |  | 93 | 15533581 | Zargar et al., | 2004 | CC | Hospital-based | Two-step | 50g 1-hour glucose level >= 7.8 mmol/L | 2,000 | 75 | 3.8  0.0 |
|  |  |  |  |  |  | WHO 1999 |  |  |  |  |  |  |
|  |  | 94 | https://www.jogi.co.in/september_october_2004/03_op_screening_for_gestati | Das et al., | 2004 | NDDG | Hospital-based | Two-step | 50g 1-hour glucose level >= 7.8 mmol/L | 300 | 12 | 4.0 |
|  |  |  | onal_diabetes_and_maternal.pdf |  |  |  |  |  |  |  |  |  |
|  | Sri Lanka | 95 | 30534375 | Dias et al., | 2018 | IADPSG | Hospital-based | One-step | N.A. | 795 | 248 | 31.2 |
|  |  | 96 | 27625764 | Herath et al., | 2016 | IADPSG | Hospital-based | One-step | N.A. | 452 | 105 | 23.2 |
|  |  | 97 | 28076942 | Sudasinghe et al., | 2016 | WHO 1999 | Community-based | One-step | N.A. | 1,400 | 194 | 13.9 |
|  |  | 98 | 12521908 | Wagaarachchi et al., | 2001 | WHO 1980 | Hospital-based | One-step | N.A. | 1,004 | 41 | 4.1 |
|  |  | 99 | 9704548 | Siribaddana et al., | 1998 | WHO 1985 | Hospital-based | Two-step | 50g 1-hour glucose level >= 7.8 mmol/L | 721 | 40 | 5.5 |
|  | Bangladesh | 100 | <https://doi.org/10.3329/jbcps.v33i2.28040> | Mustafa et al., | 2015 | Self-defined guidelines | Hospital-based | One-step | N.A. | 1,489 | 102 | 6.9 |
|  |  | 101 | 24369985 | Jesmin et al., | 2014 | WHO 1999 | Hospital-based | Two-step | 50g 1-hour glucose level >= 7.8 mmol/L | 1,149 | 112 | 9.7 |
|  |  | 102 | 16108860 | Sayeed et al., | 2005 | WHO 1999 | Community-based | One-step | N.A. | 147 | 12 | 8.2 |
|  | Nepal | 103 | 27005708 | Thapa et al., | 2015 | WHO 1999 | Hospital-based | One-step | N.A. | 564 | 14 | 2.5 |
|  |  | 104 | 22610863 | Shrestha et al., | 2011 | CC | Hospital-based | Two-step | 50g 1-hour glucose level >= 7.8 mmol/L | 1,598 | 12 | 7.5 |
|  | Pakistan | 105 | 21897896 | Jawa et al., | 2011 | IADPSG | Hospital-based | one | N.A. | 135 | 1 | 0.7 |
|  |  | 106 | 17180158 | Iqbal et al., | 2007 | ADA 2004 | Hospital-based | Two-step | 50g 1-hour glucose level >= 7.8 mmol/L | 612 | 49 | 8.0 |
|  |  | 107 | 8641127 | Akhter et al., | 1996 | WHO 1980 | Hospital-based | Two-step | 50g 1-hour glucose level >= 7.8 mmol/L | 6,830 | 223 | 3.3 |
| West Asia | Israel | 108 | 23035769 | Sella et al., | 2013 | CC | Community-based | One-step | N.A. | 367,247 | 14,288 | 3.9 |
|  |  | 109 | 21847538 | Sella et al., | 2011 | CC | Hospital-based | Two-step | 50g 1-hour glucose level >= 7.8 mmol/L | 185,315 | 11,264 | 6.1 |
|  |  | 110 | 20636958 | Chodick et al, | 2010 | CC | Community-based | One-step | N.A. | 185,416 | 11,270 | 6.1 |
|  | Iran | 111 | 30899677 | Moradi et al., | 2019 | IADPSG | Hospital-based | one-step | N.A. | 3,808 | 286 | 7.5 |
|  |  | 112 | 29649540 | Hosseini et al., | 2018 | IADPSG | Hospital-based | One-step | N.A. | 1,000 | 93 | 9.3 |
|  |  | 113 | 27350363 | Shahbazian et al., | 2016 | IADPSG | Hospital-based | One-step | N.A. | 750 | 224 | 29.9 |
|  |  | 114 | 27747467 | Pirjani et al., | 2016 | ADA 2012 | Hospital-based | One-step | N.A. | 256 | 78 | 30.5 |
|  |  | 115 | doi:10.1007/s13410-014-0209-8 | Mohammadzadeh et al., | 2015 | CC | Hospital-based | Two-step | 50g 1-hour glucose value >=7.2 mmol/L | 1,276 | 62 | 49.0 |
|  |  | 116 | 26157727 | Moradi et al., | 2015 | IADPSG | Hospital-based | One-step | N.A. | 290 | 44 | 15.2 |
|  |  | 117 | https://jfrh.tums.ac.ir/index.php/jfrh/article/view/35 | Garshasbi et al., | 2008 | CC | Hospital-based | Two-step | 50g 1-hour glucose level >= 7.2 mmol/L | 1,804 | 124 | 6.8 |
|  |  | 118 | 17962102 | Hossein-Nezhad et al., | 2007 | CC | Hospital-based | Two-step | 50g 1-hour glucose level >= 7.8 mmol/L | 2,416 | 114 | 4.7 |
|  |  | 119 | 16191491 | Hadeagh et al., | 2005 | CC | Community-based | Two-step | 50g 1-hour glucose level >= 7.8 mmol/L | 700 | 62 | 8.9 |
|  |  | 120 | 16098925 | Keshavarz et al | 2005 | CC | Hospital-based | Two-step | 50g 1-hour glucose level >= 7.8 mmol/L | 1,310 | 63 | 4.8 |
|  | Saudi Arabia | 121 | 32020147 | Alsaedi et al., | 2020 | ACOG | Hospital-based | Two-step | 50g 1-hour glucose level >= 7.8 mmol/L | 347 | 52 | 15.0 |
|  |  | 122 | 31086509 | Abualhamael et al., | 2018 | ADA 2012 | Hospital-based | one-step | N.A. | 637 | 5,000 | 12.8 |
|  |  | 123 | 29631547 | Al-Ajlan et al., | 2018 | IADPSG | Hospital-based | One-step | N.A. | 419 | 116 | 27.7 |
|  |  | 124 | 28386562 | Wahabi et al., | 2017 | WHO 2013 | Hospital-based | One-step | N.A. | 9,723 | 2,354 | 24.2 |
|  |  | 125 | ISSN 2315-6864 | Amani et al., | 2017 | IADPSG | Hospital-based | One-step | N.A. | 850 | 326 | 38.4 |
|  |  | 126 | doi:10.5603/DK.2017.0028 | Abdelmola et al., | 2017 | ADA 2014 | Hospital-based | Two-step | 50g 1-hour glucose level >= 7.8 mmol/L | 440 | 36 | 8.2 |
|  |  | 127 | 26409797 | Alfadhli et al., | 2015 | IADPSG | Hospital-based | One-step | N.A. | 573 | 292 | 51.0 |
|  |  | 128 | 25138813 | Al-Rubeaan et al., | 2014 | ADA 2011 | Community-based | One-step | N.A. | 529 | 201 | 38.0 |
|  |  | 129 | 23963090 | Wahabi et al., | 2013 | ACOG | Hospital-based | Two-step | 50g 1-hour glucose level >= 7.8 mmol/L | 3,041 | 569 | 18.7 |
|  |  | 130 | 20799591 | Al-Rowaily et al., | 2010 | WHO 1999 | Hospital-based | One-step | N.A. | 633 | 79 | 12.5 |
|  |  | 131 | 3342212 | Al-Shawaf et al., | 1998 | WHO 1985 | Hospital-based | One-step | N.A. | 1,088 | 21 | 1.9 |
|  | Qatar | 132 | 30074993 | Bashir et al., | 2018 | IADPSG | Hospital-based | One-step | N.A. | 2,000 | 430 | 21.5 |
|  |  | 133 | 22140323 | Bener et al., | 2011 | WHO 2006 | Hospital-based | One-step | N.A. | 1,608 | 262 | 16.3 |
|  |  | 134 | https://www.researchgate.net/publication/269396914_Prevalence_and_predictors_of_gestational_diabetes_in_Qatar | Al-Kuwari et al., | 2011 | ADA 2004 | Hospital-based | Two-step | 50g 1-hour glucose level >= 7.8 mmol/L | 4,295 | 275 | 6.4 |
|  | Yemen | 135 | 26869814 | Ali et al., | 2016 | ADA 2002 | Hospital-based | One-step | N.A. | 311 | 16 | 5.1 |
|  | Turkmenistan | 136 | 23867899 | Parhofer et al., | 2013 | Self-defined guidelines | Hospital-based | Two-step | 50g 1-hour glucose level >= 7.8 mmol/L | 1,620 | 109 | 6.7 |
|  | UAE | 137 | 31795984 | Hashim et al., | 2019 | NICE | Hospital-based | One-step | N.A. | 256 | 49 | 19.1 |
|  | Kuwait | 138 | 30944829 | Groof et al., | 2019 | Self-reported via questionnaire | Hospital-based | Undefined | Undefined | 868 | 109 | 12.6 |
|  | Turkey | 139 | 30641799 | Ozgu-Erdinc et al., | 2018 | NDDG | Community-based | Two-step | 50g 1-hour glucose level >= 7.8 mmol/L | 74,412 | 4,093 | 5.5 |
|  |  |  |  |  |  | IADPSG | Community-based | One-step | N.A. | 2,815 | 591 | 21.0 |
|  |  | 140 | 30402933 | Aydin et al., | 2018 | CC | Hospital-based | Two-step | 50g 1-hour glucose level >= 7.8 mmol/L | 2,643 | 428 | 16.2 |
|  |  | 141 | 28494099 | Karcaaltincaba et al., | 2017 | IADPSG | Hospital-based | One-step | N.A. | 1,434 | 159 | 11.1 |
|  |  | 142 | 26322083 | Erem et al., | 2014 | CC | Hospital-based | Two-step | 50g 1-hour glucose level >= 7.8 mmol/L | 815 | 35 | 4.3 |
|  |  | 143 | 20709580 | Karcaaltincaba et al., | 2011 | CC | Community-based | Two-step | 50g 1-hour glucose level >= 7.8 mmol/L | 1,635 | 14 | 0.9 |
|  |  |  |  |  |  |  |  |  |  |  |  |  |
|  |  | 144 | 19464683 | Karcaaltincaba et al., | 2009 | NDDG | Hospital-based | Two-step | 50g 1-hour glucose level >= 7.8 mmol/L | 21,531 | 683 | 3.2 |
|  | Bahrain | 145 | 22265190 | Rajab et al., | 2012 | NDDG | Community-based | Two-step | 50g 1-hour glucose level >= 7.8 mmol/L | 49,552 | 4,982 | 10.1 |
|  | Bahrain | 146 | 15977691 | Al Mahroos et al., | 2004 | The 4^th^ International Workshop-Conference on Gestational Diabetes Mellitus | Hospital-based | Two-step | 50g 1-hour glucose level >= 7.8 mmol/L | 10,495 | 1,417 | 13.5 |
|  | Oman | 147 | 26629376 | Abu-Heija et al., | 2015 | Self-defined guidelines | Community-based | Two-step | 50g 1-hour glucose level >= 7.8 mmol/L | 5,811 | 639 | 11.0 |

Abbreviation: CC: Carpenter-Coustan; ADA: American Diabetes Association; ACOG: American College of Obstetricians and Gynecologists; WHO: World Health Organization; NDDG: National Diabetes Data Group; IADPSG: International Association of Diabetes and Pregnancy Study Groups; DIPSI: Diabetes in Pregnancy Study Group India; ICD: International Classification of Diabetes; MOH: Ministry of Health; NICE: UK National Institute for Health and Care Excellence.
