# Supplementary material for: Gestational Diabetes Mellitus Among Asians – A Systematic Review From a Population Health Perspective
Source: Front Endocrinol (Lausanne). 2022 Jun 16;13:840331. doi: 10.3389/fendo.2022.840331 (PMC9245567; doi:10.3389/fendo.2022.840331)
Supplement: Supplementary file 7 [file DataSheet_7.docx]

**Supplementary Table 4. Diagnostic criteria for the gestational diabetes mellitus (GDM) reported in all Asian prevalence studies**

| Diagnostic criteria | PMID | OGTT | No. of abnormal | Fasting glucose, mmol/L (mg/dl) | 1-hr glucose, mmol/L (mg/dl) | 2-hr glucose, mmol/L (mg/dl) | 3-hr glucose, mmol/L (mg/dl) |
| --- | --- | --- | --- | --- | --- | --- | --- |
| CC & ADA 2005 & ACOG & ICD-10^1^ | 7148898, 16373931, 21860317, | 100g | 2 | 5.3 (95) | 10.0 (180) | 8.6 (155) | 7.8 (140) |
| NDDG | 510803 | 100g | 2 | 5.8 (104) | 10.6 (191) | 9.2 (166) | 8.0 (144) |
| Japan Diabetes Society | 11755481 | 75g | 2 | 5.5 (99) | 10.0 (180) | 8.3 (149) | -- |
| WHO 1980 | 6771926 | 75g | 1 | 7.8 (140) | -- | 11.1 (200) | -- |
| WHO 1985^2^ & WHO 1994 & DIPSI | 7941615, 16941793 | 75g | 1 | -- | -- | 7.8 (140) | -- |
| WHO 1998 & ADA 2002 | 9686693, 12502614 | 75g | 1 | 7.0 (126) | -- | 11.1 (200) | -- |
| WHO 1999^3^ | N.A. | 75g | 1 | 7.0 (126) | -- | 7.8 (140) | -- |
| WHO 2006^4^ | N.A. | 75g | 1 | 7.0 (126) | 10.0 (180) | 11.1 (200) | -- |
| ADA 2004 | 14693936 | 100g | 1 | 5.3 (95) | 10.0 (180) | 8.6 (155) | 7.8 (140) |
| ADA 2007 & the Fourth International Workshop-Conference on Gestational Diabetes Mellitus | 17192377, 9704245 | 75g | 2 | 5.3 (95) | 10.0 (180) | 8.6 (155) | 7.8 (140) |
| ADA 2011 | 21193625 | 75g | 1 | 5.1 (92) | -- | -- | -- |
| IADPSG & China MOH & ADA 2012 & ADA 2014 & WHO 2013^5^ | 20190296, 22613589, 22187469, 24357209, | 75g | 1 | 5.1 (92) | 10.0 (180) | 8.5 (153) | -- |
| Malaysia MOH^6^ & NICE^7^ | N.A. | 75g | 1 | 5.6 (101) | -- | 7.8 (140) | -- |
| Bangladesh study self-defined guidelines^8^ | N.A. | 75g | 1 | 6.1 (110) | -- | 7.8 (140) | -- |
| Turkmenistan study self-defined guidelines | 23867899 | 75g | 1 | 5.0 (90) | 10.0 (180) | 8.0 (144) | -- |
| Oman study self-defined guidelines | 26629376 | 75g | 1 | 5.5 (99) | -- | 9.0 (162) | -- |

Aberration: CC: Carpenter-Coustan; ADA: American Diabetes Association; ACOG: American College of Obstetricians and Gynecologists; WHO: World Health Organization; NDDG: National Diabetes Data Group; IADPSG: the International Association of Diabetes and Pregnancy Study Groups; DIPSI: Diabetes in Pregnancy Study Group India; ICD-10: International Classification of Diabetes; MOH: Ministry of Health; NICE: UK National Institute for Health and Care Excellence.

1. Disantostefano J. International Classification of Diseases 10th Revision (ICD-10). 2009; **5**(1): 56-7.

2. Mellitus WHOSGoD, World Health O. Diabetes mellitus : report of a WHO study group [meeting held in Geneva from 11 to 16 February 1985]. Geneva: World Health Organization; 1985.

3. World Health O. Definition, diagnosis and classification of diabetes mellitus and its complications : report of a WHO consultation. Part 1, Diagnosis and classification of diabetes mellitus. Geneva: World Health Organization; 1999.

4. World Health O, International Diabetes F. Definition and diagnosis of diabetes mellitus and intermediate hyperglycaemia : report of a WHO/IDF consultation. Geneva: World Health Organization; 2006.

5. Diagnostic Criteria and Classification of Hyperglycaemia First Detected in Pregnancy. Geneva; 2013.

6. Ministry of Health Malaysia. Perinatal Care Manual 3rd Edition. Putrajaya, Malaysia: Division of Family Health Development, MOH; 2013. 251 p.

7. Diabetes in Pregnancy: Management of Diabetes and Its Complications from Preconception to the Postnatal Period. London; 2015.

8. Mustafa F N . Pregnancy Profile and Perinatal outcome in Gestational Diabetes Mellitus: A Hospital Based Study. 2016.
