# Supplementary material for: Gestational Diabetes Mellitus Among Asians – A Systematic Review From a Population Health Perspective
Source: Front Endocrinol (Lausanne). 2022 Jun 16;13:840331. doi: 10.3389/fendo.2022.840331 (PMC9245567; doi:10.3389/fendo.2022.840331)
Supplement: Supplementary file 8 [file DataSheet_8.docx]

**Supplementary Table 5. Summary of GDM prevalence in Asia migrants**

| **No** | **PMID/DOI/**  **weblink** | **Country** | **Author** | **Year** | **GDM diagnostic method** | **Study setting** | **One-step /two-step** | **GCT-details** | **Study sample size (GDM prevalence)** |
| --- | --- | --- | --- | --- | --- | --- | --- | --- | --- |
| 1 | 33486538 | Canada | Read et al. | 2021 | ICD-10 | Population-based | Did not define | Did not define | Canadian South Asian: n=9289 (2.9%)  Canadian Chinese: n=12 240 (2.3%)  Canadian White: n=210 089 (1.4%) |
| 2 | 34059900 | Finland | Bastola et al. | 2021 | ICD-10 | Population-based | Did not define | Did not define | Finland White: n=348 313 (8.7%)  Finland South Asian: 1893 (18.4%)  Finland East Asian: 4933 (11.0%)  Finland West Asian: 2673 (12.7%) |
| 3 | 33290601 | Norway | Strandberg et al. | 2021 | WHO 1999 | Population-based | One-step | N.A. | Norwegian White: 1 116 954 (0.8%)  Norwegian South Asian: 16 506 (4.4%)  Norwegian Southeast Asian and East Asian: 32 629 (4.03%) |
| 4 | DOI: 10.1016/j.midw.2020.102899 | UK | Garcia et al. | 2021 | NICE | Population-based | One-step | N.A. | British White: n=6992 (0.4%)  British Indian: 986 (1.0%)  British Bangladeshi: 2041 (2.1%)  British Pakistani: 5123 (1.4%) |
| 5 | 32135135 | US | Li et al. | 2020 | CC | Hospital based | two-step | 50g GCT if 1-hour glucose >=7.2 mol/L | Non-Hispanic White: n=628 (4.0%); Non-Hispanic Black: n=620 (2.4%); Hispanic: n=651 (6.3%); Asian/Pacific Islander*: n=346 (7.5%) |
| 6 | 31950143 | Denmark | Neilsen et al. | 2020 | Denmark national guidelines | Hospital based | One-step | N.A. | Denmark white: n=621,154 (2.4%);  Denmark Vietnamess: n=2,633 (4.0%);  Denmark Phillipines: n=2,362 (4.8%);  Denmark Iranian: n=1,996 (5.9%);  Denmark Chinese: n=2,218 (6.5%);  Denmark Indian: n=1,132 (7.5%);  Denmark Pakistan: n=3,909 (7.1%);  Denmark Sri Lankan: n=1,673 (11.2%);  Denmark Afghanistan: n=3,281 (5.5%);  Denmark Turkish: n=8,183 (6.3%);  Denmark Iraq: n=6,150 (6.4%). |
| 7 | 31427254 | Canada | Ménard et al. | 2020 | Did not define | Hospital based | Did not define | Did not define | Canadian White: n=767 (4.2%);  Canadian South Asian: n=38 (11.1%);  Canadian East Asia-Pacific Asian: n=163 (8.8%). |
| 8 | 32075811 | Australia | Li et al. | 2020 | IADPSG | Hospital based | One-step | N.A. | Australian Asian migrants: n=491 (19.7%); Chinese Asian: n=1,000 (14.6%). |
| 9 | 30668174 | US | Liu et al. | 2019 | ICD | Hospital based | Did not define | Did not define | American white: n=6,144 (11.2%);  American Asian migrants: n=620 (24.2%);  American Arab migrants: n=1,274 (14.8%). |
| 10 | 31808419 | US | Chen et al. | 2019 | self-reported | Community based | N.A. | N.A. | Non-Hispanic White: n=1,216 (7.9%);  Asian: n= 890 (15.5%).  (Japanese: 7.9%; Korean: 9.2%; Chinese : 17.3; Vietnamese : 21.2%; Filipino: 15.0%; Indian: 24.9; Other Asian: 12.7%). |
| 11 | 30452528 | US | Williams et al. | 2019 | CC | Hospital based | two-step | 50g GCT if 1-hour glucose >=7.8 mol/L | Whites: n=4,977 (4.5%);  Blacks: n=2,148 (4.3%)  Asian/Pacific Islander*: n=899 (9.9%);  Hispanics: n=245 (6.4%);  Other races/ethnicities: n=854 (5.9%). |
| 12 | 30773821 | Australia | Wan et al. | 2019 | ICD-10 | Hospital based | One-step | N.A. | Australian White: n=28,594 (4.1%);  Australian Chinese: n=3,419 (11.6%). |
| 13 | 29501220 | US | Janevic et al. | 2018 | ICD-9 | Hospital based | Did not define | Did not define | US-born white: n=49,072 (6.1%); US-born Asian: n=72,461 (10.2%); US-Asian immigrants born foreign: n=129,279 (15.1%); US-born Chinese: n=4,121 (9.2%); US-Chinese immigrants born foreign: n=40,817 (11.6%); US-born Indian: n=9,892 (12.8%); US-Indian immigrants born foreign: n=17,766 (22.9%). |
| 14 | 26863557 | US | Sanchalika et al. | 2015 | ICD-9 | Hospital-based | Did not define | Did not define | US-born non-Hispanic white: n= 308,508 (3.5%);  Sri Lankans: n= 192 (12.5%);  Bangladeshis: n=833 (12.4%);  Indians: n= 14,612 (11.0%);  Pakistanis: n= 2,924 (10.4%). |
| 15 | 25189758 | UK | Mone et al. | 2015 | WHO 2013 | Hospital based | Did not define | Did not define | UK white: n=7,210 (1.8%);  UK-East Timorese Asian immigrants: n=42 (16.7%). |
| 16 | 26201385 | US | Pu et al. | 2015 | ICD-9 | Hospital based | Did not define | Did not define | US Non-Hispanic White: n=9,011 (7.0%); US Non-Hispanic Black: n=432 (4.9%); US Hispanic: n=3,777 (13.3%);  US Indian: n=5,069 (19.3%);  US Chinese: n=3,206 (15.3%);  US Filipino: n=1,096 (19.0%);  US Japanese: n=682 (9.7%);  US Korean: n=462 (12.9%);  US Vietnamese: n=460 (18.8%). |
| 17 | 26504462 | Australia | McDonald et al. | 2015 | Australian Diabetes in Pregnancy Society guidelines | Hospital based | One-step | N.A. | Born in Australia or New Zealand: n=1,932 (10.0%);  Born in Arab Stats: n=111 (10.8%) ;  Born in West and Central Aisa: n=138 (15.9%); Born in Southeast Asia: n=922 (17.5%);  Born in East Asia: n=154 (24.0%);  Born in South Asia: n=673 (20.2%);  Born in Africa: n=354 (9.6%);  Born in Latin America: n=41 (9.8%);  Born in Oceania n=83 (13.3%);  Born in Europe and North America: n=202 (6.9%). |
| 18 | 25679221 | Australia | Abouzeid et al. | 2015 | Gestational diabetes mellitus – management guidelines. | Hospital based | Did not define | Did not define | Born in Australia: n=209,344 (3.3%);  Born in Oceania: n=6,464 (4.1%);  Born in North-West Europe: n=9,723 (3.8%);  Born in Southern & Eastern Europe： n=6,814 (5.4%);  Born in North Africa & Middle East: n=5,470 (4.9%);  Born in South-East Asia: n=17,023 (8.4%);  Born in North-East Asia: n=7,306 (10.0%);  Born in Southern & Central Asia: n=9,338 (9.7%);  Born in Americas: n=3,472 (4.3%);  Born in Sub-Saharan Africa: n=3,695 (5.6%); Born in Australia (indigenous): n=1,596 (2.6%);  Unknown birth region: n=1,200 (3.9%). |
| 19 | 25259656 | US | Janevic et al. | 2014 | ICD-9 | Hospital based | Did not define | Did not define | Sub Saharan African: n=2,951 (4.5%);  Chinese: n=10,603 (7.2%);  South Central Asian: n=9,920 (15.8%);  Non-Hispanic Caribbean: n=16,339 (6.9%);  Dominican: n=16,423 (4.8%);  Puerto Rican: n=4,432 (5.7%);  Mexican: n=13,370 (6.8%);  Central and South American: n=20,680 (6.4%). |
| 20 | 23023978 | UK | Khalil et al. | 2013 | WHO 2006 | Hospital based | two-step | Did not define | British white: n=57,564 (reference); South Asian: n=3,645 (OR: 2.31, 95%CI: 1.90-2.80);  East Asia: n=1,793 (OR 2.26, 95% CI: 1.72-2.96). |
| 21 | 22108914 | Norway | Jenum et al. | 2012 | WHO 1999 | Community-based | One-step | N.A. | Europeans: n= 313 (reference); South Asian: n= 188 (OR 2.24; 95% CI: 1.26-3.97);  West Asian (middle east): n=112 (OR: 2.13; 95% CI: 1.12-4.08). |
| 22 | 22676578 | UK | Makgoba et al. | 2012 | Did not define | Hospital based | Did not define | Did not define | British white: n=107,906 (0.7%);  South Asian: n=15,817 (1.9%); |
| 23 | 21913963 | Australia | Wong et al. | 2011 | Australian Diabetes in Pregnancy Society guidelines | Hospital based | two-step | 50g GCT if 1-hour glucose >=7.8 mol/L | South-East Asian: n=207 (23.3%);  South Asian: n=160 (17.5%);  Middle-Eastern: n=190 (21.1%);  Pacific Islander: n=55 (27.3%);  Anglo-European: n=215 (21.4%). |
| 24 | 20155442 | US | Cripe et al. | 2011 | Did not define | Hospital based | Did not define | Did not define | Cambodian: n=3,858 (6.5%);  Laotian: n=2,223 (6.7%);  Vietnamese: n=12,949 (8.2%);  US born non-Hispanic white: n= 35,581 (4.2%). |
| 25 | 10332673 | US | Kieffer et al. | 1999 | did not define | Community based | Did not define | Did not define | Non-Hispanic white: n=6,877,668 (2.52%);  Non-Hispanic black: n=1,705,672 (2.33%);  Mexican: n=1,377,291 (2.29%); Central and South American=277,224 (2.58%);  Puerto Rican: n=158,097 (3.23%); Native American: n=107,536 (4.44%); Filipino: n=89,766 (3.99%);  Chinese: n=79,941 (4.02%);  Asian-Indian: n=37,292 (6.23%);  Cuban: n=35,821 (2.36%);  Vietnamese: n=35,039 (2.49%);  Japanese: n=36,056 (2.51%)  Korean: n=24,702 (2.09%);  Hawaiian: n=17,076 (2.93%);  Samoan: n=4,743 (2.61%). |
| 26 | 8798294 | Australia | Ma et al. | 1996 | Did not define | Hospital based | Did not define | Did not define | Australia white: n=20,049 (1.8%); Australia foreign-born Asian migrants: n=19,458 (3.6%); Australia foreign-born West Asian (middle east): n=10,171 (2.6%). |
| 27 | - 1748263 | Australia | Beischer et al. | 1991 | WHO 1985 | Hospital based | One-step | N.A. | Australian and New Zealand white: n=23, 257 (4.3%)  Vietnam: n=1300 (7.3%)  Chinese: n=653 (13.9%)  Indian: n=440 (15%) |
| 28 | 2776652 | UK | Samanta et al. | 1989 | WHO 1985 | Hospital-based | One-step | N.A. | UK white: n=504 (0.02%);  UK-Asian immigrants: n=314 (0.18%). |

Abbreviation: US: United States; UK: United Kingdom; CC: Carpenter-Coustan; WHO: World Health Organization; IADPSG: International Association of Diabetes and Pregnancy Study Groups; ICD: International Classification of Diabetes; GCT: glucose challenge test.
