# Supplementary material for: Gestational Diabetes Mellitus Among Asians – A Systematic Review From a Population Health Perspective
Source: Front Endocrinol (Lausanne). 2022 Jun 16;13:840331. doi: 10.3389/fendo.2022.840331 (PMC9245567; doi:10.3389/fendo.2022.840331)
Supplement: Supplementary file 9 [file DataSheet_9.docx]

**Supplementary Table 6. Summary table of GDM-related maternal outcome among Asian migrants**

| **Maternal Health Outcome** | **Country** | **No** | **PMID** | **Author** | **Year** | **Study design** | **Mean of follow-up (year)** | **No of GDM** | **No of outcome cases** | **Cumulative conversion rate;**  **Incidence rate (per 1000 person year) if applicable** | **Baseline age, years** | **Baseline BMI, kg/m^2^** | **GDM diagnosis guidelines** | **Outcome diagnostic guidelines** |
| --- | --- | --- | --- | --- | --- | --- | --- | --- | --- | --- | --- | --- | --- | --- |
| Prediabetes and T2D | Spain | 1 | 30309812 | Prados et al., | 2019 | Prospective cohort | 1 year | Overall: 305  Caucasian: 144  Latin American: 36  Moroccan: 29  Indian/Pakistan: 67  Chinese: 29 | Prediabetes: 112 for overall, 41 for Caucasian; 17 for Latin American; 10 for Moroccan; 29 for Indian/Pakistan and 15 for China  T2D: 16 for overall; 2 for Caucasian; 3 for Latin American; 4 for Moroccan; 7 for Indian/Pakistan; 0 for Chinese. | Prediabetes:  Overall: 36.7%;  Caucasian: 28.5%;  Latin American: 47.2%; Moroccan: 34.5%; Indian/Pakistan: 43.3%; Chinese: 51.7%;  T2D:  Overall: 5.2%;  Caucasian: 1.4%;  Latin American: 8.3%; Moroccan: 13.8%; Indian/Pakistan: 10.4%; Chinese: Nil. | Caucasian: 33.8;  Latin American 32.3;  Moroccan: 34.4;  Indian/Pakistan: 31.3;  China: 32.8 | Caucasian: 27.6;  Latin American 28.4;  Moroccan: 28.7; Indian/Pakistan: 27.3;  China: 23.6 | NDGG | ADA 2010 |
|  | US | 2 | 22526611 | Mekerji et al., | 2012 | Prospective cohort study | 7.6 years | Overall (Chinese, South Asian and White): 33203 | Did not specify | T2D:  China: 16.5%, 25.8;  South Asia: 31.8%, 54.7;  White: 25.7%, 39.7. | Chinese: 32.0;  South Asian: 29.0;  White: 30.0 | Did not mention | ICD | ICD |

Abbreviation: T2D: type 2 diabetes; GDM: gestational diabetes mellitus; BMI: body mass index; ICD: International Classification of Diseases; ADA: American Diabetes Association; US: United States
