# Supplementary material for: Gestational Diabetes Mellitus Among Asians – A Systematic Review From a Population Health Perspective
Source: Front Endocrinol (Lausanne). 2022 Jun 16;13:840331. doi: 10.3389/fendo.2022.840331 (PMC9245567; doi:10.3389/fendo.2022.840331)
Supplement: Supplementary file 10 [file DataSheet_10.docx]

**Supplementary Table 7. Summary table of maternal GDM-related offspring health outcomes among Asian migrants**

| **Offspring outcomes** | **No** | **PMID** | **Country** | **Author** | **Year** | **Study design** | **Mean or range of follow-up** | **Total offspring number of outcomes** | **Baseline maternal age & offspring age** | **Multiple variable adjustment** | **Effect size** |
| --- | --- | --- | --- | --- | --- | --- | --- | --- | --- | --- | --- |
| **Fetal outcomes** | | | | | | | | | | | |
| **Anthropometry** | 1 | 28253366 | Norway | Slenter et al. | 2017 | Prospective cohort | N.A. | Whites: 39 born to GDM mothers (total n=349);  South Asian: 28 born to GDM mothers (total n=184). | Mom: Did not mention  Fetus: weeks 24 | Maternal parity and fetal gender | Fetuses borne by GDM mothers tended to be smaller than fetuses borne by non-GDM mothers in week 24, but thereafter grew faster until birth. This pattern was especially pronounced in fetuses of South Asian mothers with moderate/severe GDM. In week 24 these fetuses had a -0.95 SD (95% CI: -1.53, -0.36) lower estimated fetal weight than their non-GDM counterparts, and yet had a larger birthweight of 0.45 SD (0.09, 0.81) at birth. |
| **Neonatal outcomes** | | | | | | | | | | | |
| **Anthropo-metry** | 2 | 28800568 | Canada | Anand et al. | 2016 | prospective cohort | at birth | NHW: 51 out of 387 born to GDM mothers;  South Asian: 84 out of 401 born to GDM mothers | Mom: 29.7-31.2 years  Offspring: newborn | Gestational age, sex and insulin use by mother during pregnancy. | Among full-term newborns, South Asians had lower birthweight (3283 vs 3517 g, p = 0.0001), had greater skinfold thickness (11.7 vs 10.6 mm; p=0.0001) and higher waist circumference (31.1 vs 29.9 cm, p=0.0001) compared with NHW. |
|  | 3 | 26819358 | Nether-lands | Kosman et al. | 2016 | Prospective cohort | at birth | Macrosomia: 64 out of 387 children born to GDM mothers  (Whites: 33; Turkish: 8; others: 23) | Mom: 30.6-33.4 years  Offspring: newborn | Crude model | Caucasian children born to GDM mothers had higher birthweight (3482.7 vs 3469.0), higher rate of macrosomia (22.6% vs. 18.6%), compared with Turkish children born to GDM mothers. |
|  | 4 | 23571827 | US | Bower et al. | 2013 | Retrospective cohort study | at birth | LGA: n=891 born to GDM mothers (n=4853); n=9798 born to non-GDM mothers (n=101132)  Macrosomia: n=511 born to GDM mothers (n=4853); n=7207 born to non-GDM mothers (n=101132).  Did not stratify the racial/ethnic percentage in each group. | Mom: 28.4 years  Offspring: newborn | Crude model | GDM were independently associated with increased risk of LGA infant across all races (except for NWH). Across all races, having GDM, obesity and high pregnancy weight gain joint effect on odd of LGA was substantially increased in NHW (OR: 11.27, 8.40-15.11), in non-Hispanic black (7.09, 4.81-10.45) and in Hispanic (10.19, 6.84-15.19), respectively. However, for API, the joint effect of all three factors (5.14, 2.11-12.50) was approximately the same as any of the two factors. |
|  | 5 | 21365298 | US | Mocarski et al.3 | 2012 | Retrospective cohort study | at birth | 19 416 out of 536 084 children born to GDM mother  NHW: 3,349  Chinese: 1,528  African American: 2,479  Non-Chinese API: 548; South Central Asian: 2,777  Others: 8 735 | Mom:  <25 years: 30.1%;  25-30: years 26.6%;  30-35 years: 25.3%;  35-40 years: 14.2%;  ≥40 years: 3.8%  Offspring: newborn | Maternal age, being foreign born, insurance, education, parity, tobacco use during pregnancy, and pre-pregnancy weight. | Newborns born to GDM mothers tended to have higher risk of preterm birth and macrosomia in both non-Hispanic white and US-Chinese migrants, with similar effect size. |
| **Child outcomes** | | | | | | | | | | | |
| **Anthro-pometry** | 6 | 30855657 | US | Faith et al. | 2019 | prospective cohort | 2-5 years | 382 children born to GDM mothers  NHW: 130  NHB: 26  Hispanic: 126  Asia: 96  Others: 4 | Mom: 33.3 years  Offspring: 56.5 months | OGTT a-score, maternal ppBMI, maternal age and race/ethnicity, birth order (parity), infant weight-for-length z score at birth, sum of breastfeeding intensity and duration ratios from birth through 12 months of age, and age at initiation of 100% fruit juice/sugar-sweetened beverage intake during the first year. | Children born to GDM mothers with a high soothability temperament in infancy had a 2.2-fold to 2.5-fold increased odds of future obesity at 2-5 years of age, across all races/ethnicities. |
|  | 7 | 29064033 | UK | West et al. | 2018 | Prospective cohort | 4-5 years | White British: 187 out of 2717 born to GDM mothers;  Pakistani: 529 out of 3343 born to GDM mothers | Mom: Did not mention  Offspring: 4-5 years | Offspring sex and age at measurement and maternal age, parity, BMI, SES and smoking | There was no evidence that maternal gestational fasting glucose, post-load glucose or a diagnosis of GDM were positively associated with offspring adiposity in either ethnic group in this study. |
|  | 8 | 26217524 | UK | Fairley et al. | 2015 | prospective cohort | 3 years | British White: 27 out of 382 born to GDM mother;  Pakistani: 65 out of 474 born to GDM mothers | Mom: did not mention  Offspring: 3 years | Maternal age & highest educational qualification, parity, infant ethnicity & sex, GA at delivery, birthweight, and mode of delivery. | GDM did not seem to affect 3-year offspring BMI z-score in both ethnicities. |

Abbreviation: GDM: gestational diabetes mellitus; BMI: body mass index; LGA: large for gestational age; SD: standard deviation; SE: standard error; CI: confident interval; OR: odds ratios; US: United states; UK: United Kingdom.
